# Using In Vitro and In Silico Analysis to Investigate the Chemical Profile and Biological Properties of *Polygonum istanbulicum* Extracts

**DOI:** 10.3390/plants13233421

**Published:** 2024-12-05

**Authors:** Giancarlo Angeles Flores, Gaia Cusumano, Gokhan Zengin, Mehmet Veysi Cetiz, Abdullahi Ibrahim Uba, Ismail Senkardes, Ismail Koyuncu, Ozgur Yuksekdag, Alina Kalyniukova, Carla Emiliani, Roberto Venanzoni, Paola Angelini

**Affiliations:** 1 Department of Chemistry, Biology and Biotechnology, University of Perugia, 06123 Perugia, Italy; giancarlo.angelesflores@unich.it (G.A.F.); gaia.cusumano@dottorandi.unipg.it (G.C.); carla.emiliani@unipg.it (C.E.); roberto.venanzoni@unipg.it (R.V.); 2 Botanic Garden “Giardino dei Semplici”, Department of Pharmacy, “Gabriele d’Annunzio” University, 66100 Chieti, Italy; 3 Department of Biology, Science Faculty, Selcuk University, Konya 42130, Turkey; 4 Department of Medical Biochemistry, Faculty of Medicine, Harran University, Sanliurfa 63290, Turkey; mvcetiz@gmail.com (M.V.C.); ismailkoyuncu1@gmail.com (I.K.); ozguryuksekdag47@gmail.com (O.Y.); 5 Department of Molecular Biology and Genetic, Faculty of Science and Arts, Istanbul Arel University, Istanbul 34537, Turkey; abdullahi.iu2@gmail.com; 6 Department of Pharmaceutical Biology, Pharmacy Faculty, Marmara University, Istanbul 34722, Turkey; isenkardes@marmara.edu.tr; 7 Faculty of Forestry and Wood Sciences, Czech University of Life Sciences Prague, Kamýcká 129, 165 21 Prague, Czech Republic; kalyniukova@gmail.com

**Keywords:** *Polygonum istanbulicum*, antioxidant, quercetin, myricetin, enzyme inhibitory, cytotoxic, molecular dynamics

## Abstract

The present study investigates the chemical profile and biological activities of *Polygonum istanbulicum* M. Keskin, a species endemic to Turkey, aiming to explore its potential applications in pharmacology. We assessed its phenolic and flavonoid content by employing ethyl acetate, methanol, and water as extraction solvents. The methanol extract demonstrated the highest concentrations of these compounds, with liquid chromatography–quadrupole time-of-flight tandem mass spectrometry (LC-MS-qTOF) analysis identifying a wide range of bioactive substances, such as derivatives of quercetin and myricetin. Antioxidant capacity was evaluated using 2,2-Diphenyl-1-picrylhydrazyl (DPPH), 2,2′-Azino-bis(3-ethylbenzothiazoline-6-sulfonic acid) diammonium salt (ABTS), cupric-reducing antioxidant capacity (CUPRAC), ferric-reducing antioxidant power (FRAP), and phosphomolybdenum assays, with the methanol extract showing the most potent activity (DPPH: 892.22 mg Trolox equivalent (TE)/g; ABTS: 916.21 mg TE/g; CUPRAC: 1082.69 mg TE/g; FRAP: 915.05 mg TE/g). Enzyme inhibition assays highlighted the efficacy of *P. istanbulicum* extracts against key enzymes, with potential implications for managing Alzheimer’s disease, hyperpigmentation, and type 2 diabetes. Cytotoxicity tests against various cancer cell lines showed notable activity, particularly with the aqueous extract on the HGC-27 cell line (IC_50_: 29.21 µg/mL), indicating potential for targeted anti-cancer therapy. Molecular docking and molecular dynamics simulations further supported the binding affinities of quercetin and myricetin derivatives to cancer-related proteins, suggesting significant therapeutic potential. This study underscores the value of *P. istanbulicum* as a source of bioactive compounds with applications in antioxidant, anti-cancer, and enzyme-inhibitory treatments.

## 1. Introduction

Since ancient times, plants with medicinal properties have played a fundamental role in human health and have always been used in traditional medicine worldwide [1]. Even today, using plants for medicinal purposes is very common, particularly due to the belief that plants (and products derived from them), being part of nature, are safer than synthetic medicines [2]. In recent decades, interest in medicinal plants has increased significantly, both in the search for new pharmacologically active compounds and due to the growing resistance to existing synthetic drugs [3]. Studies conducted in recent years have highlighted the therapeutic potential of various phytocompounds in treating chronic diseases such as cancer, diabetes, and cardiovascular disorders [4,5]. Flavonoids and phenolic acids, critical components of medicinal plants, exhibit significant antioxidant and anti-inflammatory properties, essential for preventing oxidative stress and related diseases [6]. Medicinal plants as therapeutic agents, which are increasingly attracting researchers’ attention worldwide, also offer various advantages over conventional medicines, such as low costs, wide availability, and fewer side effects [7]. Currently, many species of medicinal plants are used not only for their therapeutic effects but also in cosmetics as colorants and in the food industry as flavoring agents [8,9].

Among medicinal plants, those belonging to the *Polygonum* genus have been used for centuries to treat various disorders [10,11]. The *Polygonum* genus belongs to the Polygonaceae family. It includes about 300 annual and perennial species found worldwide and growing in different climates, from northern temperate regions to tropical and subtropical areas [12,13]. In 2009, within the *Polygonum* genus, a new species, *P. istanbulicum* M. Keskin, was described by Keskin [14]. *P. istanbulicum* is a narrow endemic species found in Istanbul, Turkey. Several species of this genus have already been investigated for their therapeutic potential. Among the most well-known species of this genus are *P. aviculare* and *P. hydropiper*. Idoudi et al. [10] summarized the uses of these two species. In Portugal, *P. aviculare* is known for its laxative and diuretic effects, while in Morocco, this species is used for treating digestive disorders, kidney stones, and hyperglycemia. In India, the leaf powder of *P. aviculare* is used for urinary tract diseases and infections, whereas *P. hydropiper* is used for cardiac disorders, hypertension, hemorrhoids, and intestinal conditions. Other species of the *Polygonum* genus that have already been investigated include *P. amphibium*, *P. arenarium*, *P. barbatum*, *P. bellardii*, *P. bistorta*, *P. capitatum*, *P. cognatum*, *P. cuspidatum*, *P. equisetiforme*, *P. flaccidum*, *P. glabrum*, *P. hyrcanicum*, *P. lapathifolium*, *P. maritimum*, *P. multiflorum*, *P. orientale*, *P. persicaria*, *P. plebeium*, *P. tortuosum*, and *P. viviparum* [10,15,16,17,18], demonstrating biological properties such as antioxidant, anti-inflammatory, antidiabetic, antimicrobial, and antitumor activities. The literature reports that the *Polygonum* genus contains various compounds, including phenolic acids such as gallic, caffeic, and *p*-coumaric acids and flavonoids like quercetin, catechin, and kaempferol. It also contains tannins, stilbenes (notably polydatin and resveratrol), terpenes (such as α-pinene, β-caryophyllene, and β-farnesene), and various fatty acids like oleic, palmitic, and stearic acids. Quercetin, in particular, is well known for its properties, including anti-cancer, antiviral, anti-inflammatory, and cardiovascular effects [10,19,20,21].

The biological properties of *P. istanbulicum* have not been described; therefore, the current study is the first on this species. The aim of this work is to analyze the chemical profile of the species in question, evaluate its phenol and flavonoid content, and also assess its biological properties in terms of antioxidant activity, enzyme inhibition, and cytotoxicity against normal and cancer cells. The obtained results can provide new information about the biological properties of *P. istanbulicum* and open new avenues for its possible use in pharmaceutical, nutraceutical, and cosmeceutical formulations.

## 2. Results and Discussion

### 2.1. Total Phenolic and Flavonoid Content

Phenolic compounds and flavonoids are essential phytochemicals in plants that significantly contribute to their antioxidant activities and offer potential benefits for human health. The Folin–Ciocalteu and AlCl_3_ assays are widely used to determine total phenolic content and flavonoid content, respectively, in plant extracts and provide initial insights into potential antioxidant capacity [22,23]. This study assessed the total phenolic content (TPC) and the total flavonoid content (TFC) of the extracts of *P. istanbulicum*. The results are reported in Table 1. In terms of total phenolic content, the best extract was methanol, with a value of 108.20 ± 1.40 mg gallic acid equivalent (GAE)/g, followed by water (80.14 ± 0.83 mg GAE/g) and then ethyl acetate (22.07 ± 0.11 mg GAE/g). In terms of total flavonoid content, methanol was the best, with a value of 37.01 ± 0.38 mg rutin equivalent (RE)/g; water and ethyl acetate exhibited values of 16.41 ± 1.12 mg RE/g and 15.78 ± 0.45 mg RE/g, respectively. In the literature, no studies have been reported on this species’ phenol and flavonoid content. The work by Abdullah et al. [24] on ethyl acetate, methanol, and water extracts of *P. minus* leaves showed that the highest phenol and flavonoid content was found in the methanolic extract. Another study by Hanif et al. [25] on dichloromethane and methanolic extracts of *P. glabrum* showed that the highest phenol and flavonoid content was found in the methanolic extract. Although conducted on other species, these results seem consistent with our findings.

### 2.2. Liquid Chromatography–Quadrupole Time-of-Flight Tandem Mass Spectrometry (LC-MS-qTOF) Analysis

**Figure 1:**
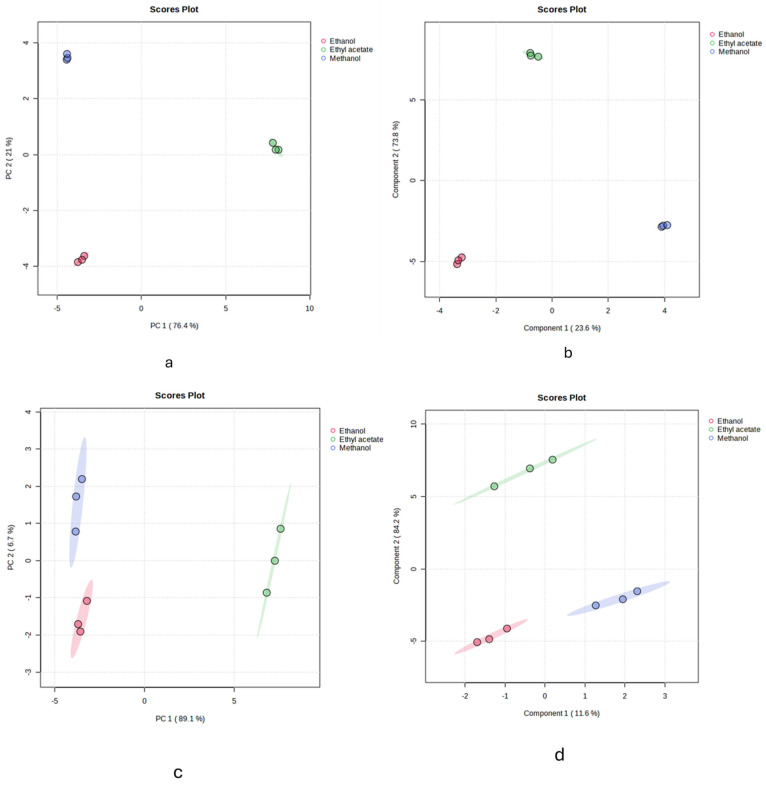
(**a**) PCA scores of *Polygonum istanbulicum* extracts in negative ionization mode. (**b**) PCA scores of *Polygonum istanbulicum* extracts in positive ionization mode. (**c**) PLSDA analysis of *Polygonum istanbulicum* extracts in negative ionization mode. (**d**) PLSDA analysis of *Polygonum istanbulicum* extracts in positive ionization mode.

The Principal Component Analysis (PCA) is a powerful tool for transforming correlated variables into linearly independent components (principal components) through orthogonal transformation. This facilitates a deeper understanding of the data structure and the relationships between variables. In this study, an unsupervised PCA model was built to analyze the similarities and differences between three groups obtained using three different solvents: methanol, ethanol, and ethyl acetate. Figure 1 presents the PCA score plots for two processing methods in both positive (A) and negative (B) ion modes. The results show that the samples extracted using different solvents are classified into distinct categories in both positive and negative ion modes, indicating significant differences in the composition and concentration of components when using different extraction solvents.

**Figure 2:**
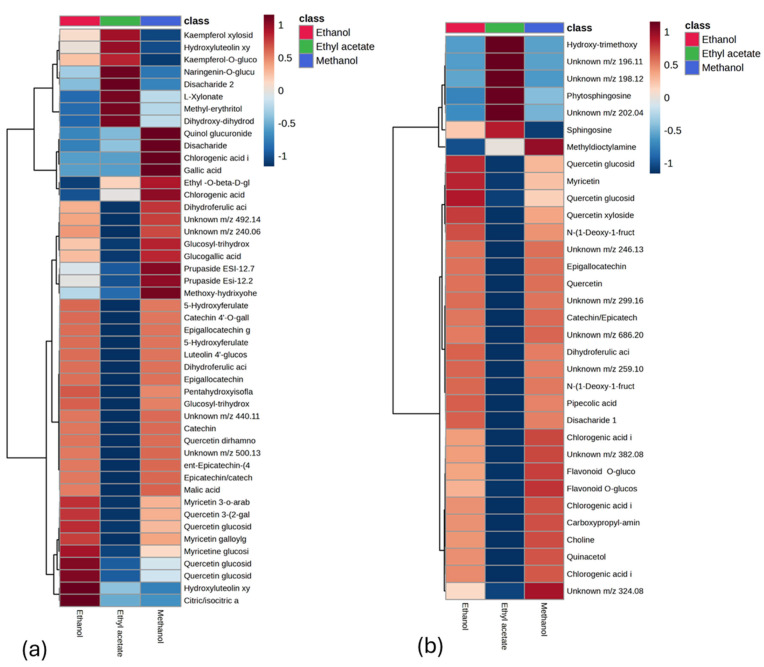
Heatmap of *Polygonum istanbulicum* extracts in negative (**a**) and positive (**b**) ionization mode.

A total of 83 features were detected across both positive and negative ion modes, with 50 features identified in negative ionization mode and 33 in positive ionization mode. The results are presented in Table 2 and Table 3. To determine the key components responsible for the differences among the three groups, a supervised Partial Least Squares Discriminant Analysis (PLS-DA) model was developed (Figure 1b,c). This model facilitated the identification of the differential components that significantly contributed to the separation between the groups. Based on VIP values greater than 1.5 and *p* < 0.05, a heatmap analysis was performed to visually represent the changes in compound composition across samples extracted with the three solvents, following multivariate statistical analysis (Figure 2). The results demonstrate significant differences in the overall chemical composition of the samples depending on the solvent used for extraction. For instance, methanol and ethanol proved more effective at extracting most polyphenols and flavonoids, yielding similar results, while ethyl acetate showed comparatively lower extraction efficiency for these compounds.

### 2.3. Antioxidant Effects

Assessing the antioxidant activity of plant extracts is a critical step in transforming natural resources into pharmaceutical products, as it allows the identification of bioactive compounds capable of preventing oxidative stress-related diseases. Therefore, understanding the antioxidant capacities derived from plants is essential for developing drugs targeting disorders associated with oxidative stress, which are often linked to various chronic diseases [26]. In this research, we examined the scavenging activity using the 2,2-Diphenyl-1-picrylhydrazyl (DPPH) and 2,2′-Azino-bis(3-ethylbenzothiazoline-6-sulfonic acid) diammonium salt (ABTS) assays, assessed the reducing power through the cupric-reducing antioxidant capacity (CUPRAC), ferric-reducing antioxidant power (FRAP), and phosphomolybdenum methods, and analyzed the metal-chelating capacity of the different extracts of *Polygonum istanbulicum*. The results are shown in Table 4. In the DPPH assay, the best scavenging capacity was observed for the methanol and water extracts (892.22 ± 11.52 mg TE/g and 835.79 ± 25.58 mg Trolox equivalent (TE)/g, respectively). The ethyl acetate extract exhibited a low value (13.32 ± 0.56 mg TE/g) compared to the other extracts. In the ABTS assay, the methanol extract showed the highest activity (916.21 ± 22.84 mg TE/g), followed by the water extract, with a value of 786.25 ± 15.34 mg TE/g. In this assay, the ethyl acetate extract showed the lowest scavenging capacity, with a value of 37.61 ± 4.54 mg TE/g.

Regarding the reducing capacity tests, in the CUPRAC assay, the highest ability was observed in the methanol extract (1082.69 ± 75.57 mg TE/g). In contrast, the water extract had a value of 688.95 ± 5.88 mg TE/g, and the ethyl acetate extract had 85.30 ± 2.96 mg TE/g. Similarly, in the FRAP assay, the methanol extract demonstrated the best reducing performance, with a value of 615.05 ± 8.64 mg TE/g. The water extract had a value of 360.35 ± 7.17 mg TE/g, and the ethyl acetate extract was the lowest, at 36.55 ± 0.18 mg TE/g. The phosphomolybdenum assay, which relies on the reduction of Mo (VI) to Mo (V) by antioxidant compounds in acidic conditions, has recently emerged as a popular method for evaluating the reducing power of extracts, primarily due to its straightforwardness and the absence of a need for specialized equipment. In this assay, the methanol extract again showed the highest reducing capacity, with a value of 3.47 ± 0.16 mmol TE/g. The values obtained for the water and ethyl acetate extracts were 2.31 ± 0.01 mmol TE/g and 2.04 ± 0.13 mmol TE/g, respectively. The chelation of transition metals is an important antioxidant mechanism that helps prevent the formation of hydroxyl radicals during the Fenton reaction, thereby reducing oxidative stress. In this study, the most potent chelating ability was observed for the ethyl acetate extract (12.09 ± 0.41 mg EDTA equivalent (EDTAE)/g), followed by the water extract (9.10 ± 0.83 mg EDTAE/g) and then the methanol extract (5.99 ± 0.95 mg EDTAE/g). Observing the results obtained in these assays, it is notable that the methanol extracts were the most effective in almost every assay, particularly in scavenging and reducing assays. On the other hand, the least activity was observed for the ethyl acetate extracts, except for its chelating ability, which was the highest compared to the methanol and water extracts. No studies on the antioxidant power of *P. istanbulicum* have been reported in the literature. Other species have been investigated; for example, a study on the antioxidant properties using DPPH, ABTS, and FRAP assays was conducted on ethyl acetate, methanol, and water extracts of *Polygonum minus* leaves, revealing that the methanolic extract showed the best antioxidant activity across all tested assays [24]. These results are not comparable to ours as they were expressed in μmol TE/g extract. Another study by Hanif et al. [25] on dichloromethane and methanolic extracts of *P. glabrum* showed that the methanolic extract exhibited the best antioxidant ability across all tested assays, with results expressed in mg TE/g extract. Their results cannot be compared with ours since different species, extraction methods, and plant parts were used. In summary, it can be stated that the methanolic extract showed the best antioxidant capacity in these studies as well as in our study

### 2.4. Enzyme-Inhibitory Effects

Natural enzyme inhibitors play a fundamental role in managing non-infectious diseases such as Alzheimer’s, hyperpigmentation, and type 2 diabetes by acting on key enzymes involved in these pathologies. For instance, acetylcholinesterase (AChE) inhibitors treat Alzheimer’s disease because they help maintain higher levels of acetylcholine in the brain [27]. Similarly, tyrosinase inhibitors manage hyperpigmentation disorders by preventing melanin synthesis. In contrast, α-amylase and α-glucosidase inhibitors are effective in reducing blood glucose levels, thereby playing a significant role in controlling type 2 diabetes [28]. In this study, the inhibitory effects of methanol, ethyl acetate, and water extracts of *P. istanbulicum* were evaluated against the enzymes AChE, butrylcholinesterase (BChE), tyrosinase, amylase, and glucosidase. As reported in Table 5, against AChE, the methanol extract showed the best inhibition capacity, with a value of 2.54 ± 0.06 mg galantamine equivalent (GALAE)/g; the ethyl acetate extract showed a value of 2.17 ± 0.28 mg GALAE/g, and water was the weakest (1.97 ± 0.05 mg GALAE/g). The ethyl acetate extract was the best against BChE, with a value of 3.21 ± 0.22 mg GALAE/g, followed by water and methanol, with values of 2.23 ± 0.15 mg GALAE/g and 1.99 ± 0.32 mg GALAE/g, respectively. Regarding the tyrosinase inhibition assay, the most potent extract was methanol (70.15 ± 1.00 mg kojic acid equivalent (KAE)/g), followed by water (52.85 ± 0.54 mg KAE/g), and ethyl acetate (34.40 ± 1.63 mg KAE/g). Against amylase, the inhibition activity was low, with values of 0.62 ± 0.01 mmol acarbose equivalent (ACAE)/g, 0.55 ± 0.02 mmol ACAE/g, and 0.39 ± 0.03 mmol ACAE/g for the water, ethyl acetate, and methanol extracts, respectively. In the glucosidase assay, water and methanol showed values of 1.06 ± 0.01 mmol ACAE/g and 1.02 ± 0.02 mmol ACAE/g, respectively. For the ethyl acetate extract, no inhibition against glucosidase was observed (Table 5). There are no studies on the enzymatic inhibition capacity of *P. istanbulicum*. Studies have been conducted on other species. The study by Ahmad et al. [29] revealed significant inhibitory properties of *P. minus* against AChE and BChE; these findings are not comparable to ours, as the values were expressed in IC50 and the solvents used for extraction were different. In the study by Saleem et al. [30] on the enzymatic inhibition capacity of *P. plebeium* extracts, the results for AChE, BChE, tyrosinase, α-amylase, and α-glucosidase were expressed as mg GALAE/g extract, mg KAE/g extract, and mmol ACAE/g extract, respectively. These results are not comparable to ours since the extracts were not prepared in the same way. The results obtained in this study highlight that *P. istanbulicum* appears to have potential enzymatic inhibition capacity.

### 2.5. Cytotoxicity

Extracts of *P. istanbulicum* obtained using ethyl acetate, methanol, and water were employed to evaluate their cytotoxic effects against five different cancer cell lines: HGC-27 (gastric adenocarcinoma), MDA-MB-231 (human breast cancer), HELA (cervical cancer), HT-29 (human colorectal adenocarcinoma), and DU-145 (human prostate cancer). The cytotoxicity assay was conducted using the IC_50_ method (50% Inhibitory Concentration), which determines the concentration of extract required to inhibit cell growth by 50%. The results, shown in Table 6, are presented as IC_50_ values in μg/mL. For the HGC-27 cell line, the aqueous extract exhibited the highest efficacy with an IC_50_ of 29.21 μg/mL, followed by the methanolic extract (62.96 μg/mL) and, lastly, the ethyl acetate extract (92.11 μg/mL). This indicates that the active compounds against this cell line may be more water-soluble. In the MDA-MB-231 cell line, the aqueous extract once again demonstrated the highest activity (IC_50_ 51.05 μg/mL), followed by the ethyl acetate extract (56.23 μg/mL), and finally, the methanolic extract (72.48 μg/mL). The difference between the extracts was less pronounced in this case. For HELA cells, the methanolic extract proved to be the most effective (IC_50_ 57.12 μg/mL), followed by the water and ethyl acetate extracts, with values of 85.31 μg/mL and 113.80 μg/mL, respectively. In the HT-29 cell line, the aqueous extract once again showed the highest activity (IC_50_ 45.20 μg/mL), followed by the ethyl acetate extract (60.01 μg/mL) and the methanolic extract (65.82 μg/mL). Finally, for the DU-145 cell line, the methanolic extract showed the highest efficacy (IC_50_ 38.09 μg/mL), followed by the aqueous extract (33.28 μg/mL) and the ethyl acetate extract (93.48 μg/mL). Comparing the extracts, it is evident that the aqueous extract tends to be more effective across most cell lines, particularly against HGC-27. The methanolic extract showed superior activity against HELA and DU-145, whereas the ethyl acetate extract generally appeared less effective, with the exception of the MDA-MB-231 cell line, where it displayed greater efficacy. This study revealed that the highest sensitivity to *P. istanbulicum* extracts was observed in the HGC-27 cell line with the aqueous extract, showing an IC50 value of 29.21 μg/mL. This suggests the potential of aqueous extracts of *P. istanbulicum* for a targeted therapeutic approach to this type of cancer cell. On the other hand, the lowest sensitivity was observed for the HELA cell line with the ethyl acetate extract, with an IC50 value of 113.80 μg/mL, making this extract potentially appropriate for applications that require less harmful effects. There are no studies in the literature regarding the cytotoxic effect of *P. istanbulicum*. This has been evaluated for other species of the *Polygonum* genus, such as *P. minus*. Rohin et al. [31] tested various extracts of *P. minus* for their cytotoxic effects on different cell types, including HT-29 cells, which were also used in the present study. The results of this study, expressed as IC50 (µg/mL), showed that the ethyl acetate extract had an excellent cytotoxic effect when compared to the methanolic and aqueous extracts; however, these results are not comparable with ours as they refer to a different species. In another study by Baghi [32], the cytotoxic effect of the methanolic extract of *P. avicular* was tested on HELA cells, highlighting this species’ cytotoxic potential. Again, the results are not comparable with ours, even though the methanolic extract had a better cytotoxic effect on HELA cells in our study. Our results and findings from other studies suggest that species within the *Polygonum* genus may be exploited as potential sources for developing novel drugs against various types of cancer.

### 2.6. Evaluating Docking Outcomes: Ligand-Binding Energies and Interaction Profiles

This study employed a comprehensive evaluation to investigate the antimicrobial properties of compounds identified in *P. istanbulicum* against specific cancer-related proteins, including MDA-MB-231 (EGFR, ARO, PI3K delta, CDK4, CDK2, AKT-1, BCL-2, BCL-W, MCL-1, TGF-β1), HeLa (EGFR, ARO, PI3K delta, CDK4, CDK2, AKT-1, BCL-2, BCL-W, MCL-1, TGF-β1), HGC-27 (EGFR, ARO, PI3K delta, CDK4, CDK2, AKT-1, BCL-2, BCL-W, MCL-1, TGF-β1), HT-29 (EGFR, ARO, PI3K delta, CDK4, CDK2, AKT-1, BCL-2, BCL-W, MCL-1, TGF-β1), and DU-145 (EGFR, ARO, PI3K delta, CDK4, CDK2, AKT-1, BCL-2, BCL-W, MCL-1, TGF-β1), along with standard enzymes (AChE, BChE, Tyr, amylase, and glucosidase). Table S1 shows the coordinates and grid sizes used in the analyses. Chemical profiling identified numerous beneficial compounds such as hydroxyluteolin and its derivatives, myricetin and its derivatives, quercetin glucoside and its derivatives, and chlorogenic acid and its isomers. In particular, the analysis included the following chemicals: 3,4,5,7-tetramethoxyflavone, quercetin 4-O-glucoside, quercetin 3-O-xyloside, 5-caffeoylquinic acid, myricetin 3-O-rhamnoside, 4-caffeoylquinic acid, quercetin 3-O-rutinoside, quercetin 3-O-arabinoside, quercetin 3-O-galactoside, myricetin 3-O-rutinoside, laricitrin, myricetin 3-O-glucoside, chlorogenic acid, 3,5-dicaffeoylquinic acid, myricetin, quercetin 3-O-rhamnoside, myricetin 3-beta-O-galactoside, quercetin 3-O-glucoside, hydroxyluteolin, syringetin, and 3,4-dicaffeoylquinic acid. The study’s main objective was to investigate the standard enzymatic and anti-cancer properties of these phytochemicals in the context of conventional enzymes and proteins associated with cancer. Compounds with binding energies less than −9 kcal/mol are highlighted in Table 1 and those with higher energies are listed in Table S2**.** The docking data revealed a range of binding energies from −11.2 to −4.2 kcal/mol (Table S2).

**Figure 3:**
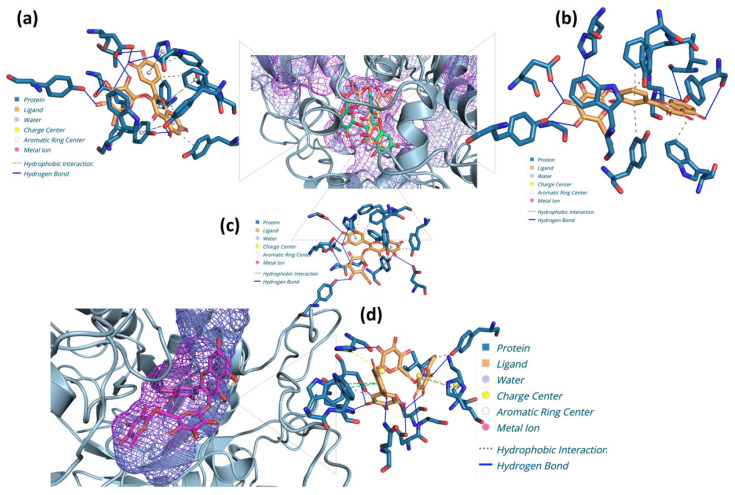
Enzyme and protein active sites with compounds showing the best binding energy. (**a**) Interaction between AChE and quercetin 3-O-rhamnoside. (**b**) Interaction between AChE and quercetin 4-O-glucoside. (**c**) Interaction between AChE and quercetin 3-O-galactoside. (**d**) Interaction between BChE and myricetin 3-O-rutinoside.

The results of the molecular docking study indicated the presence of strong inhibitory effects on proteins associated with cancer development. The results demonstrated strong binding affinities for these target proteins with quercetin derivatives, including quercetin 3-O-rhamnoside, quercetin 4-O-glucoside, quercetin 3-O-rutinoside, quercetin 3-O-arabinoside, and quercetin 3-O-galactoside. Additionally, the study identified the presence of quercetin 3-O-xyloside and myricetin derivatives, including myricetin 3-O-rhamnoside, myricetin 3-O-rutinoside, myricetin 3-O-glucoside, and myricetin 3-beta-O-galactoside (Table S3). The root mean square deviation (RMSD), which represents the accuracy of ligand binding within the protein’s active site, constituted a pivotal element of the docking analysis. Higher RMSD values may indicate less precise ligand placement, whereas lower values typically indicate more dependable binding interactions. In contrast to AChE, the binding energies of the compound quercetin 3-O-rhamnoside, quercetin 4-O-glucoside, and quercetin 3-O-galactoside were robust, with corresponding RMSD values of 1.05 Å, 0.15 Å, and 0.60 Å, and values of −11.2, −11.0, and −11.0 kcal/mol, respectively. The formation of 11, 8, and 11 hydrogen bonds, respectively, indicated the stability of these ligands within the active region of the enzyme and supported these interactions. Similarly, myricetin 3-O-rutinoside demonstrated a high binding affinity for BChE, with a binding energy of −10.8 kcal/mol and an RMSD value of 0.79 Å, forming 10 hydrogen bonds, which suggest a stable interaction (Figure 3).

These findings highlight the potential of the investigated phytochemicals as potent inhibitors of standard enzymes and cancer-related proteins. Notably, these compounds exhibit significant inhibitory effects on a range of proteins linked to cancer, including CDK2, PI3K delta, AKT, Eg5, TOP2A, Casp-3, ARO, Traf2, CDK4, IL-2, and EGFR. Among the key findings, 3,5-dicaffeoylquinic acid demonstrated strong inhibition against AKT with a binding energy of −10.3 kcal/mol, formed nine hydrogen bonds, and showed an RMSD value of 0.39 Å. Similarly, myricetin 3-O-rutinoside showed potent activity against CDK2, with a binding energy of −9.5 kcal/mol, forming 14 hydrogen bonds, and showing an RMSD of 0.45 Å. Both myricetin 3-O-rutinoside and myricetin 3-O-rhamnoside exhibited robust inhibitory activity against EGFR, with binding energies of −10.2 kcal/mol and −10.0 kcal/mol, and RMSD values of 1.09 Å and 0.90 Å, respectively. Furthermore, quercetin 3-O-arabinoside was identified as a potent inhibitor of CDK4, displaying a binding energy of −10.1 kcal/mol, forming 12 hydrogen bonds with CDK4, and showing an RMSD value of 1.06 Å, indicating a stable interaction within the enzyme’s active site. Quercetin 3-O-rutinoside was also identified as a strong inhibitor of aromatase (ARO), with a binding energy of −10.6 kcal/mol, forming 10 hydrogen bonds with ARO, and showing an RMSD of 0.88 Å, suggesting a reliable and stable binding configuration within the enzyme’s binding pocket. Additionally, quercetin 3-O-rutinoside demonstrated efficacy against Traf2 with a binding energy of −10.0 kcal/mol, forming six hydrogen bonds, and showing an RMSD value of 1.03 Å, further underscoring the compound’s potential anti-cancer properties. In conclusion, this study illustrates the multifaceted inhibitory potential of phytochemicals derived from *Polygonum istanbulicum*, highlighting their ability to interact stably with both conventional enzymes and cancer-related proteins. These findings suggest their significant promise as therapeutic agents in cancer treatment and enzyme inhibition, warranting further exploration in medicinal applications.

### 2.7. Binding Free Energy Analysis: Molecular Mechanics—Poisson–Boltzman Surface Area (MM/PBSA) Results and Implications for Ligand Efficacy

This study evaluated the impact of energy components on binding stability by calculating and analyzing a series of protein–ligand complexes. The investigation focused on a number of important energy parameters, including van der Waals interaction (VDWAALS), electrostatic energy (EEL), polar solvation energy (EGB), surface tension (ESURF), gas-phase energy (GGAS), solvation energy (GSOLV), and total energy (TOTAL). The assessment of cancer-related enzyme activities of anthraquinone derivatives derived from *Polygonum istanbulicum* was conducted through the utilization of MM/PBSA binding free energy calculations in conjunction with molecular dynamics simulations. Six complexes were selected for further analysis based on factors including low root mean square deviation (RMSD), high binding energy, and the quantity of hydrogen bonds produced. The complexes selected for further analysis are as follows: The following complexes were selected for additional analysis: EGFR_myricetin 3-O-rhamnoside, EGFR_myricetin 3-O-rutinoside, AKT_3,5-Dicaffeoylquinic acid, ARO_quercetin 3-O-rutinoside, ARO_myricetin 3-O-rutinoside, and CDK4_quercetin 3-O-arabinoside (Table S4).

**Figure 4:**
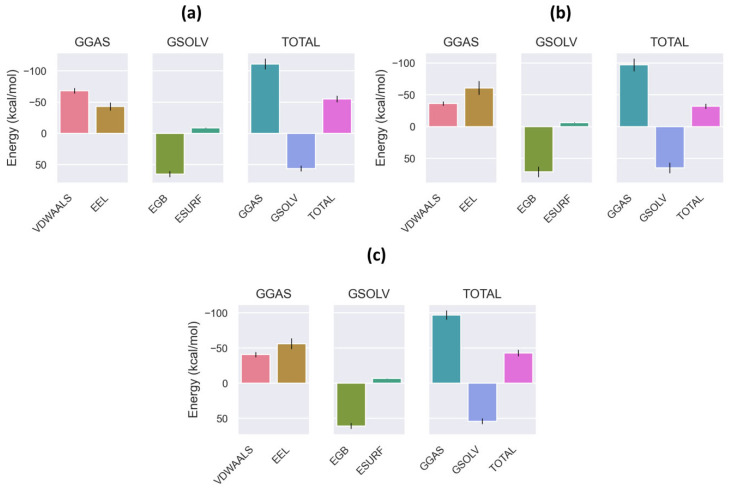
MM/PBSA binding free energy analysis. (**a**) ARO_quercetin 3-O-rutinoside complex. (**b**) CDK4_quercetin 3-O-arabinoside complex. (**c**) EGFR_myricetin 3-O-rhamnoside complex.

As illustrated in Figure 4, three complexes stand out as exhibiting exceptional stability with regard to binding. The complexes exhibiting the most promising binding stability are ARO_quercetin 3-O-rutinoside, EGFR_myricetin 3-O-rhamnoside, and CDK4_quercetin 3-O-arabinoside. These complexes are indicated with a red highlight in Table S3. It is noteworthy that the electrostatic energy value of −56.25 kcal/mol and the polar solvation energy of 60.71 kcal/mol in the EGFR_myricetin 3-O-rhamnoside complex indicate particularly strong binding stability, as indicated by the total energy value of −43.07 kcal/mol. In consideration of binding affinity, two additional complexes are worthy of particular mention. These are ARO_quercetin 3-O-rutinoside, which exhibits a binding energy of −54.83 kcal/mol, and CDK4_quercetin 3-O-arabinoside, which displays a binding energy of −32.00 kcal/mol. In comparison to the other complexes, the AKT_3-5-Dicaffeoylquinic acid complex exhibits diminished binding stability, with a total energy of −21.05 kcal/mol (Table S3). In conclusion, these findings demonstrate that these compounds possess robust binding affinities to protein targets, suggesting their potential as inhibitor candidates and their significant contribution to drug development research.

### 2.8. Stability and Flexibility in Molecular Dynamics Simulation

This study aims to identify potential therapeutic agents through a comprehensive analysis of the molecular interactions between selected ligands and target proteins, with a particular focus on elucidating their binding sites. Three ligands were selected for evaluation based on key parameters, including the presence of hydrogen bonding residues, the results of MM/PBSA binding free energy calculations, and the scores obtained from molecular docking. The selected ligand–protein complexes, namely CDK4 with quercetin 3-O-arabinoside, EGFR with myricetin 3-O-rhamnoside, and ARO with quercetin 3-O-rutinoside, exhibited robust selectivity and stability in their interactions. These complexes were subjected to molecular dynamics (MD) simulations to further assess their potential as therapeutic agents, which provided deeper insights into their biological efficacy and protein-binding capabilities.

**Figure 5:**
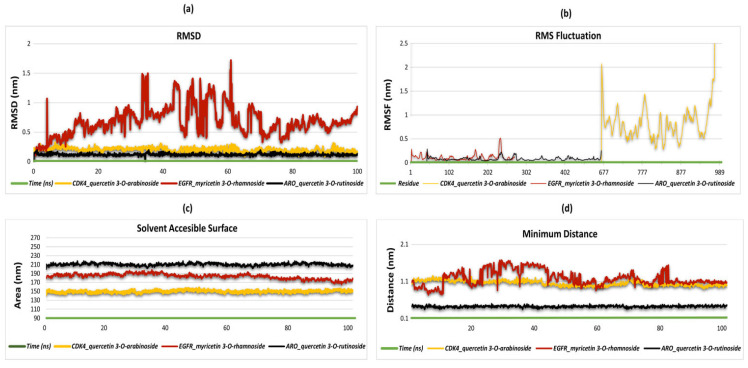
Presentation of molecular dynamics simulations in graphical form; (**a**) RMSD of CDK4_quercetin 3-O-arabinoside. EGFR_myricetin 3-O-rhamnoside and ARO_quercetin 3-O-rutinoside complexes. (**b**) RMSF of CDK4_quercetin 3-O-arabinoside. EGFR_myricetin 3-O-rhamnoside and ARO_quercetin 3-O-rutinoside complexes. (**c**) Solvent accessibility of AChE_quercetin 3-O-glucoside. BChE_quercetin 3-O-rhamnoside-7-O-glucoside. glucosidase-kaempferol 3,6-acetylglucoside-7-rhamnoside, and tyrosinase-quercetin 3-caffeoylrobinobioside complexes. (**d**) Minimum distance of CDK4_quercetin 3-O-arabinoside. EGFR_myricetin 3-O-rhamnoside and ARO_quercetin 3-O-rutinoside complexes.

The RMSD (root mean square deviation) graph demonstrates the structural stability and dynamic behavior of the three ligand–protein complexes throughout the simulation. The EGFR_myricetin 3-O-rhamnoside complex exhibited notable fluctuations and elevations in RMSD values, particularly between 10 ns and 70 ns, indicating structural flexibility and dynamic movement in the binding region. This suggests that the ligand and protein interactions are less stable and exhibit greater mobility. In contrast, the complexes of CDK4 with quercetin 3-O-arabinoside and ARO with quercetin 3-O-rutinoside exhibited lower RMSD values, approximately 0.5 nm and 0.2–0.3 nm, respectively, indicating higher structural stability. These lower RMSD levels demonstrate that both complexes underwent minimal structural changes during the simulation and that the ligands formed strong and consistent interactions with the proteins. In general, the EGFR_myricetin 3-O-rhamnoside complex exhibited greater structural flexibility, whereas the CDK4_quercetin 3-O-arabinoside and ARO_quercetin 3-O-rutinoside complexes demonstrated enhanced stability and stronger interactions. These findings offer valuable insights into ligand–protein interactions’ structural stability and dynamic nature (Figure 5a). The RMSF (Root Mean Square Fluctuation) graph provides a comparative analysis of the flexibility of residues in the three ligand–protein complexes throughout the simulation. The EGFR_myricetin 3-O-rhamnoside and ARO_quercetin 3-O-rutinoside complexes exhibit high RMSF values, indicating that the residues in their respective binding regions are highly stable. In contrast, the CDK4_quercetin 3-O-arabinoside complex displays a notable increase in flexibility, particularly in the vicinity of residue 677, indicating enhanced dynamicity in this region. In general, the stability and flexibility of the binding regions differ between the complexes. The CDK4_quercetin 3-O-arabinoside complex displays greater mobility and flexibility, while the other two complexes exhibit more stable structures (Figure 5b). The SASA (solvent accessible surface area) graph presents a comparative analysis of the surface areas exposed to the solvent in the three ligand–protein complexes. The CDK4_quercetin 3-O-arabinoside complex exhibits a surface area ranging from 145 to 155 nm^2^, while the EGFR_myricetin 3-O-rhamnoside complex maintains a surface area between 180 and 200 nm^2^. The ARO_quercetin 3-O-rutinoside complex exhibits the highest surface area, ranging from 210 to 220 nm^2^, with a stable trend throughout the simulation. These findings indicate that the solvent-accessible surface areas vary between the complexes, which could impact the nature of their protein–ligand interactions (Figure 5c). The minimum distance graph compares the shortest distances between atoms in the three ligand–protein complexes. The complexes of CDK4_quercetin 3-O-arabinoside and EGFR_myricetin 3-O-rhamnoside exhibit elevated mobility, with minimum distances within the range of 0.2 to 1.7 nm. This suggests that the binding regions of these ligands display greater flexibility. In contrast, the ARO_quercetin 3-O-rutinoside complex consistently exhibits lower distances, ranging between 0.3 and 0.5 nm, which suggests a stronger and more stable interaction. The graph illustrates the disparities in mobility and interaction strength within the binding regions of the complexes (Figure 5d).

**Figure 6:**
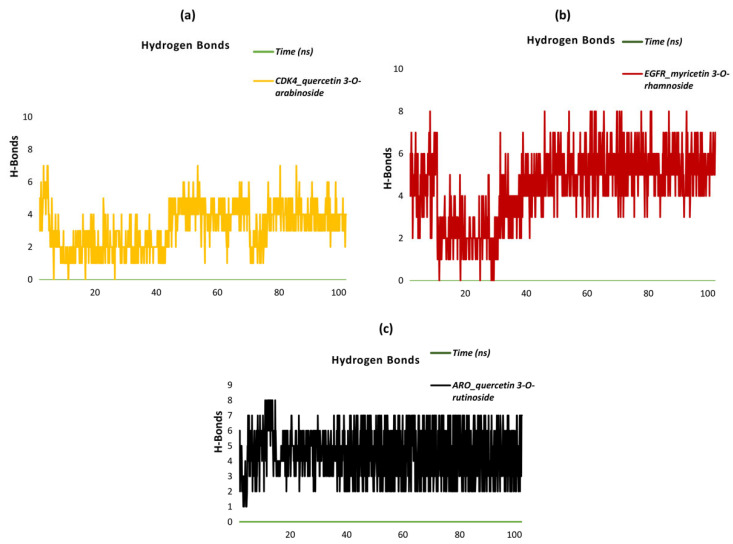
Hydrogen bonds in complexes. (**a**) Hydrogen bonds in CDK4_quercetin 3-O-arabinoside. EGFR_myricetin 3-O-rhamnoside and ARO_quercetin 3-O-rutinoside complexes. (**b**) Hydrogen bonds in CDK4_quercetin 3-O-arabinoside. EGFR_myricetin 3-O-rhamnoside and ARO_quercetin 3-O-rutinoside complexes. (**c**) Hydrogen bonds in CDK4_quercetin 3-O-arabinoside. EGFR_myricetin 3-O-rhamnoside and ARO_quercetin 3-O-rutinoside complexes.

The dynamics of the hydrogen bonds (H-bonds) across the three ligand–protein complexes, CDK4_quercetin 3-O-arabinoside, EGFR_myricetin 3-O-rhamnoside, and ARO_quercetin 3-O-rutinoside, demonstrate distinct trends throughout the simulation (Figure 6a–c). In the CDK4_quercetin 3-O-arabinoside complex, there was a notable variation in the number of hydrogen bonds over the initial 40 nanoseconds, with a range of two to seven bonds observed. Subsequently, the number of hydrogen bonds stabilizes between three and five. In the latter stages of the simulation, particularly after 75 ns, an increase in the formation of hydrogen bonds is observed, with the number fluctuating between five and seven. This indicates that the binding region exhibits enhanced stability over time. A comparable pattern is observed in the EGFR_myricetin 3-O-rhamnoside complex. Substantial fluctuations are evident during the initial 30 ns, with bond numbers oscillating between two and eight. Subsequently, the hydrogen bonds stabilize, exhibiting fluctuations between three and seven. It is noteworthy that after 50 ns, there is a slight increase, with the bond count stabilizing between four and eight. This trend indicates that the interactions within the binding region become increasingly stable over time, with hydrogen bonds playing a pivotal role in maintaining the structural integrity of the complex. In the case of the ARO_quercetin 3-O-rutinoside complex, the formation of hydrogen bonds is observed at an early stage of the simulation, with the number of bonds varying between two and seven over the initial 40 nanoseconds. Subsequently, the number of hydrogen bonds exhibits stability, fluctuating between three and seven bonds for the remainder of the simulation. It is noteworthy that after 60 ns, the bond count remains relatively stable, thereby supporting the notion that hydrogen bonds are pivotal for maintaining the long-term stability of the complex’s binding region. The analysis demonstrates that the formation of hydrogen bonds is a crucial factor in the stability of all three complexes, with each complex exhibiting an initial phase of fluctuation followed by a period of stabilization. As the simulation progresses, particularly after 60 ns, the hydrogen bond counts in all three complexes indicate a more stable interaction, thereby reinforcing the importance of hydrogen bonding in maintaining the integrity of these ligand–protein complexes.

## 3. Materials and Methods

### 3.1. Plant Collection

**Figure 7:**
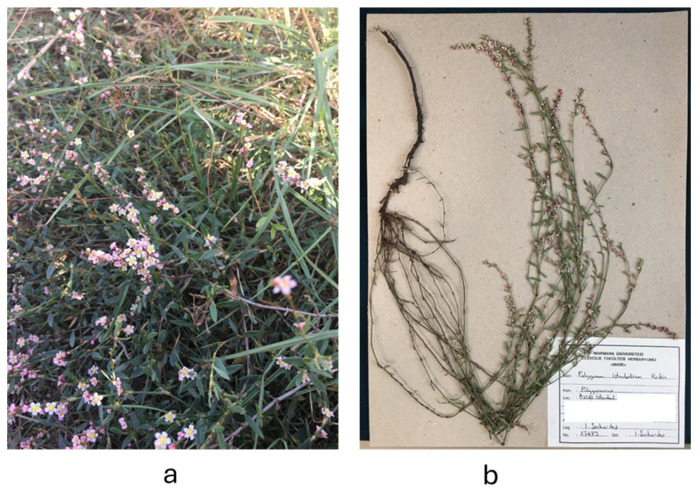
*Polygonum istanbulicum*. (**a**) Plant in natural habitat; (**b**) herbarium specimen.

In 2024, botanical specimens of *P. istanbulicum* M. Keskin [14,33] were collected from the Maltepe Başıbüyük area in Istanbul, Turkey. Dr. Ismail Senkardes conducted the taxonomic identification, and a voucher specimen was preserved in the herbarium of the Pharmacy Faculty at Marmara University (voucher number: MARE-23489). The photograph and herbarium record are also given in Figure 7. The aerial parts were segregated, dried in the shade at ambient temperature, pulverized, and stored away from light.

### 3.2. Plant Extract Preparation

The extraction procedure included three solvents: ethyl acetate, methanol, and water. Each 10 g sample from aerial parts of this plant was macerated with 200 mL of ethyl acetate and methanol for 24 h at ambient temperature. The aqueous extract was prepared by infusing 10 g of plant material in boiling water for 15 min. Organic solvents were removed via evaporation under low pressure, and the aqueous extract was subjected to freeze-drying.

### 3.3. Assay for Total Phenolic and Flavonoid Contents

Total phenolics and flavonoids were quantified according to the procedures outlined by [34]. Gallic acid (GA) and rutin were used as reference standards in the studies, with results expressed as gallic acid equivalent (GAE) and rutin equivalent (RE).

### 3.4. LC-MS-Q-TOF Metabolomic Analysis

Metabolomic analysis was conducted using an Agilent 1290 Infinity II system in conjunction with an Agilent 6546 LC/MS QTOF apparatus (Agilent, Santa Clara, CA, USA). Separation was accomplished utilizing the InfinityLab Poroshell 120 EC-C18 column (2 × 150 mm, 2.7 µm) manufactured by Agilent (USA). All analytical information is provided in the Supplementary Materials.

### 3.5. Assays for In Vitro Antioxidant Capacity

Following the methodologies detailed in our prior publication [35], various antioxidant tests were carried out. The outcomes were represented as milligrams of Trolox equivalent (TE) per gram for the DPPH and ABTS radical-scavenging, CUPRAC, and FRAP tests. The phosphomolybdenum (PBD) test examined antioxidant potential in millimoles of Trolox equivalent (TE) per gram of extract, and the metal-chelating activity (MCA) was determined in milligrams of disodium edetate equivalent (EDTAE) per gram of extract.

### 3.6. Inhibitory Effects Against Some Key Enzymes

Following the established protocols [35], experiments on enzyme inhibition were performed on the samples. Acarbose equivalent (ACAE) per gram of extract was used to measure the activities that inhibit amylase and glucosidase. In contrast, milligrams of galanthamine equivalent (GALAE) per gram of extract was used to examine the inhibition of acetylcholinesterase (AChE) and butyrylcholinesterase (BChE). The amount of tyrosinase inhibition for each gram of extract was measured in milligrams of kojic acid equivalent (KAE).

### 3.7. Cytotoxic Evaluation

#### 3.7.1. Cell Culture

This study utilized cancer and normal cell lines acquired from ATCC and preserved in liquid nitrogen. DU-145 (Prostate Carcinoma), MDA-MB-231 (Breast Adenocarcinoma), HELA (Cervix Adenocarcinoma), HT-29 (Colon Adenocarcinoma), HCT-116 (Colorectal Carcinoma), A549 (Lung Adenocarcinoma), HGC-27 (Gastric Carcinoma), and HEK-293 (Embryonic Kidney Epithelial) cells were cultivated in DMEM-F12/RPMI-1640 media enriched with 10% Fetal Bovine Serum (FBS) and 100 μg/mL of streptomycin/100 IU/mL of penicillin in incubators at 37 °C under humidified conditions with 5% CO_2_.

#### 3.7.2. Cell Viability Assay

The cytotoxic effects of the extracts were evaluated utilizing the MTT (3-(4,5-Dimethylthiazol-2-yl)-2,5-Diphenyltetrazolium Bromide) test. The cells (DU-145, MDA-MB-231, HELA, HT-29, HCT-116, A549, HGC-27, and HEK-293) were cultured in a sterile 96-well plate for 24 h at a density of 1 × 10^4^ cells per well. The medium was eliminated, and the extracts were incubated at concentrations of 0, 2.5, 5, 10, 25, 50, 100, and 200 μg/mL for a duration of 24 h. Then, 10 μL of MTT (0.5 mg/mL) was applied to each well as the reactive agent. Following a 4 h incubation, the material was discarded and replaced with 100 μL of DMSO, following which optical density measurements were conducted at OD570-OD690nm utilizing a plate reader (Thermo Multiskan GO, Thermo, Waltham, MA, USA). Subsequent to these measurements, plots were generated and the IC50 value was determined.

#### 3.7.3. Molecular Docking Protocol for Ligand–Protein Binding Analysis

The proteins and enzymes utilized in this study were procured from the Protein Data Bank (PDB). The pertinent details are presented in Table S1. Following the retrieval of the structures, the co-crystallized ligands, cofactors, and water molecules were meticulously removed using BIOVIA Discovery Studio Visualizer V4.5 in order to prepare the proteins for biochemical experimentation. This step was of great importance in order to guarantee that the proteins were suitable for docking studies. The ligands were optimized using OpenBabel V3.1.1 subsequent to retrieval from the PubChem database [36,37]. Furthermore, additional preparation of the protein and enzyme structures was conducted using MGL Tools version 1.5.6 to guarantee their suitability for subsequent analysis. The active sites of these proteins were identified using the CavitOmiX V1.0 plugin in PyMOL V2.5.8 or through literature-based inhibitor-binding sites (Table S1) [38,39]. To validate the precision of the preliminary docking outcomes, a re-docking procedure was conducted. The protein was re-docked with the ligand, and the accuracy of the docking was evaluated by calculating the root mean square deviation (RMSD) values. The following formula was employed to calculate RMSD, which is a metric that quantifies the mean discrepancy between the atomic coordinates of the reference and target structures:

$$\mathrm{RMSD}=\sqrt{\frac{1}{N}\sum_{i=1}^{N}{\left(r_{i}^{\mathrm{ref}}-r_{i}^{\mathrm{target}}\right)}^{2}}$$

The validation of protein–ligand interactions was conducted using PLIP software version 2021, with a particular emphasis on hydrogen bond interactions. Molecular docking was performed with AutoDock Vina V1.1.2, and the grid boxes were configured in accordance with the methodology outlined by Trott and Olson [40].

#### 3.7.4. MM/PBSA Free Energy Calculations to Assess Ligand-Binding Affinity

In this study, the gmx_MMPBSA tool (https://valdes-tresanco-ms.github.io/gmx_MMPBSA/dev/getting-started/ (accessed on 1 November 2024)) was employed to calculate the free energy and evaluate the stability of the compounds. Based on the results of the 10-nanosecond molecular dynamics (MD) simulations, the most stable compounds were selected for further analysis. Subsequently, extended molecular dynamics simulations (100 ns) were conducted on the selected compounds [41,42].

#### 3.7.5. Molecular Dynamics Simulation Setup for Ligand Stability and Flexibility

Molecular dynamics (MD) simulations were initiated using the CHARMM Graphical User Interface (GUI) platform, accessible at https://charmm-gui.org (accessed on 1 November 2024). The system setup was performed with the Solution Builder tool, originally developed by Jo et al. [43]. The CHARMM36m force field was employed for protein parameterization, in accordance with the protocols delineated by Yagi et al. [44] and Maier et al. [45]. A periodic boundary box was filled with TIP3P water molecules, ensuring that the minimum distance between the protein and the box edges was 10 Å. To achieve a neutral system and adjust the NaCl concentration to 0.15 M, counterions were introduced. The handling of electrostatic and van der Waals interactions was conducted using the Verlet cutoff scheme, while bond lengths were constrained via the LINCS algorithm. The particle mesh Ewald (PME) method was employed for the calculation of long-range electrostatics. Energy minimization was conducted using the steepest descent algorithm until the potential energy reached a value below 1000 kJ/mol/nm. Subsequently, the system was equilibrated under NVT and NPT ensembles at 303.3 K to ensure thermodynamic stability. The production simulations were conducted using GROMACS 2023.2 for a total duration of 100 ns (nstep = 50,000,000).

#### 3.7.6. Statistical Analysis

The experiments were executed in triplicate, and differences among the extracts were assessed using an ANOVA and Tukey’s test. The statistical analysis was conducted using Graph Pad Prism (version 9.2).

## 4. Conclusions

In summary, the present study is the first report on the chemical profiles and biological effects of the different extracts from *P. istanbulicum*. LC-MS-QTOF analysis revealed the presence of some pharmacologically important compounds such as derivatives of quercetin and myricetin. The methanol extract showed the best radical-scavenging and reducing abilities. The methanol and water extracts also had great cytotoxic potential in the cell lines tested. The present study demonstrates the robust binding affinity and stability of quercetin and myricetin derivatives with cancer-associated proteins, highlighting their potential as therapeutic agents. The results suggest that these compounds could play a crucial role in the development of new drugs and in the treatment of cancer. Based on these findings, *P. istanbulicum* can be considered a valuable source of health-promoting compounds in the preparation of functional applications. However, further studies are strongly recommended to understand the pharmacological mechanism of individual compounds from *P. istanbulicum*.

## Figures and Tables

**Table 1:** Total phenolic and flavonoid contents in the tested extracts *.

Extracts	TPC (mg GAE/g)	TFC (mg RE/g)
Ethyl acetate	22.07 ± 0.11 ^c^	15.78 ± 0.45 ^b^
Methanol	108.20 ± 1.40 ^a^	37.01 ± 0.38 ^a^
Water (infused)	80.14 ± 0.83 ^b^	16.41 ± 1.12 ^b^

* Values are reported as mean ± SD of three parallel measurements. GAE: gallic acid equivalent; RE: rutin equivalent. Different letters indicate significant differences between the tested extracts (*p* < 0.05).

**Table 2:** Chemical composition of the tested extracts in negative ionization mode.

	Compound	Compound Group	Formula	Mass	Retention Time. min	Ion Species
1	Methyl-erythritol-phosphate	Phosphate Derivative	C5H13O7P	216.0405	1.30	[M-H]^−^
2	Dihydroxy-dihydrodibenzothiophene	Phosphate Derivative	C12H10O2S	218.0374	1.31	[M-H]^−^
3	Xylonate	Sugar Acid	C5H7O5	166.0479	1.31	[M-H]^−^
4	Disaccharide	Carbohydrate	C12H22O11	378.0934	1.34	[M-H]^−^
5	Disaccharide 2	Carbohydrate	C12H22O11	342.1162	1.35	[M-H]^−^
6	Malic acid	Dicarboxylic Acid	C4H6O5	134.0216	1.53	[M-H]^−^
7	Citric/isocitric acid	Tricarboxylic Acid	C4H6O5	192.0271	1.90	[M-H]^−^
8	Glucogallic acid	Phenolic Compound	C14H10O7	332.0745	2.20	[M-H]^−^
9	Glucosyl-trihydroxyacetophenone isomer 1	Flavonoid	C18H22O7	330.0951	2.79	[M-H]^−^
10	Gallic acid	Phenolic Compound	C7H6O5	170.0216	2.96	[M-H]^−^
11	Epigallocatechin	Flavonoid	C15H14O7	306.0743	4.17	[M-H]^−^
12	Procyanidin B	Flavonoid	C30H26O13	578.1424	6.33	[M-H]^−^
13	Methoxy-hydrixyohenylglycol glucuronide	Glycoside	C18H20O10	360.1061	6.46	[M-H]^−^
14	Cinnamtannin A	Flavonoid	C45H36O18	866.2052	6.56	[M-H]^−^
15	Unknown *m*/*z* 492.1482			492.1482	6.69	[M-H]^−^
16	Quinol glucuronide	Glycoside		286.0691	6.88	[M-H]^−^
17	Glucosyl-trihydroxyacetophenone isomer 2	Flavonoid	C18H22O7	330.0957	7.51	[M-H]^−^
18	Hydroxyferulate	Phenolic Compound	C10H10O5	210.053	7.51	[M-H]^−^
19	Catechin	Flavonoid	C15H14O7	290.0792	7.7	[M-H]^−^
20	Unknown *m*/*z* 392.21319			392.1316	8.32	[M-H]^−^
21	Chlorogenic acid	Flavonoid	C16H18O9	354.095	8.330	[M-H]^−^
22	Ethyl-O-ß-D-glucopyranosyl-butanoate	Glycoside	C15H20O8	294.1317	8.99	[M-H]^−^
23	Chlorogenic acid isomer	Flavonoid	C16H18O9	354.0951	9.11	[M-H]^−^
24	Epigallocatechin gallate	Flavonoid	C22H18O11	458.0852	9.39	[M-H]^−^
25	Unknown *m*/*z* 500.1353			500.1353	10.06	[M-H]^−^
26	Epicatechin/catechin 10.094001	Flavonoid	C15H14O7	290.0793	10.09	[M-H]^−^
27	Unknown *m*/*z* 440.1147			440.1147	10.59	[M-H]^−^
28	Unknown *m*/*z* 240.067			240.067	10.71	[M-H]^−^
29	Dihydroferulic acid-O-glucuronide isomer 1	Glycoside	C16H16O7	372.1063	10.84	[M-H]^−^
30	Dihydroferulic acid-O-glucuronide isomer 2	Glycoside	C16H16O7	372.106	11.09	[M-H]^−^
31	Quercetin di-deoxyhexoside.	Flavonoid	C21H20O11	594.1584	11.24	[M-H]^−^
32	Catechin-O-gallate	Flavonoid	C22H20O10	442.0903	11.69	[M-H]^−^
33	Myricetin galloylglucoside	Flavonoid	C26H24O11	632.1019	12.17	[M-H]^−^
34	Prupaside isomer 2	Flavonoid	C27H28O10	552.2209	12.22	[M-H]^−^
35	Hydroxyferulate 12.444	Phenolic Compound	C10H10O5	210.053	12.44	[M-H]^−^
36	Prupaside isomer 2	Flavonoid	C27H28O10	552.2201	12.76	[M-H]^−^
37	Myricetin hexoside	Flavonoid	C24H24O10	480.0905	13.59	[M-H]^−^
38	Quercetin-galloyl hexoside	Flavonoid	C21H20O11	616.106	14.26	[M-H]^−^
39	Luteolin-hexoside-pentoside.	Flavonoid	C22H20O12	624.1326	14.92	[M-H]^−^
40	Quercetin hexoside isomer 1	Flavonoid	C21H20O11	464.0961	15.26	[M-H]^−^
41	Myricetin O-pentoside.	Flavonoid	C20H18O12	450.0813	15.27	[M-H]^−^
42	Quercetin hexoside isomer 2	Flavonoid	C21H20O12	464.0959	16.12	[M-H]^−^
43	Quercetin hexoside isomer 3	Flavonoid	C21H20O12	464.0958	16.46	[M-H]^−^
44	Hydroxyluteolin O-pentoside isomer 1	Flavonoid	C26H28O15	434.0851	17.45	[M-H]^−^
45	Hydroxyluteolin O-pentoside isomer 2	Flavonoid	C26H28O15	434.085	18.46	[M-H]^−^
46	Hydroxyluteolin O-pentoside isomer 3	Flavonoid	C26H28O15	434.0853	18.46	[M-H]^−^
47	Kaempferol O-hexoside.	Flavonoid	C21H20O11	448.1006	18.91	[M-H]^−^
48	Naringenin-O-glucuronide	Flavonoid	C21H20O11	448.1007	19.14	[M-H]^−^
49	Kaempferol-O-pentoside	Flavonoid	C20H18O10	418.09	20.81	[M-H]^−^
50	Pentahydroxyisoflavone	Flavonoid	C15H10O7	302.0428	21.93	[M-H]^−^

**Table 3:** Chemical composition of the tested extracts in positive ionization mode.

	Compound	Compound Group	Formula	Mass	Retention Time. min	Ion Species
1	Choline	Quaternary Amine	C5H14NO	103.0996	1.24	[M+H]^+^
2	Carboxypropyl-amino-deoxyfructose	Amino Sugar	C12H21NO6	265.116	1.29	[M+H]^+^
3	Disaccharide 1	Carbohydrate	C12H22O11	341.1329	1.30	[M+H]^+^
4	Unknown *m*/*z* 202.0451			202.0451	1.30	[M+H]^+^
5	Unknown *m*/*z* 259.1055			259.1055	1.38	[M+H]^+^
6	N-(1-Deoxy-1-fructosyl)proline	Amino Acid Derivative	C11H17N1O5	277.1161	1.38	[M+H]^+^
7	Pipecolic acid	Amino Acid	C6H11NO2	129.0789	1.52	[M+H]^+^
8	N-(1-Deoxy-1-fructosyl)valine	Amino Acid Derivative	C11H19N1O5	279.1316	1.61	[M+H]^+^
9	Unknown *m*/*z* 299.169			299.1369	2.03	[M+H]^+^
10	Unknown *m*/*z* 324.0828			324.0828	3.08	[M+H]^+^
11	Epigallocatechin	Flavonoid	C15H14O7	306.0734	4.18	[M+H]^+^
12	Chlorogenic acid isomer 1	Flavonoid	C16H18O9	354.0924	4.46	[M+H]^+^
13	Unknown *m*/*z* 686.2031			686.2031	4.46	[M+H]^+^
14	Unknown *m*/*z* 382.088			382.088	6.47	[M+H]^+^
15	Unknown *m*/*z* 246.1368			246.1368	6.48	[M+H]^+^
16	Unknown *m*/*z* 187.0634			187.0634	6.65	[M+H]^+^
17	Catechin/Epicatechin	Flavonoid	C15H14O7	290.0792	7.72	[M+H]^+^
18	Chlorogenic acid isomer 1	Flavonoid	C16H18O9	354.0959	8.34	[M+H]^+^
19	Chlorogenic acid isomer 2	Flavonoid	C16H18O9	354.095	9.12	[M+H]^+^
20	Flavonoid O-glucoside	Flavonoid		574.2019	12.23	[M+H]^+^
21	Dihydroferulic acid-O-glucuronide	Flavonoid	C16H16O7	372.1054	12.46	[M+H]^+^
22	Flavonoid O-hexoside isomer 1	Flavonoid		574.2017	12.77	[M+H]^+^
23	Unknown *m*/*z* 196.1101			196.1101	12.81	[M+H]^+^
24	Myricetin	Flavonoid	C15H10O7	318.0375	15.30	[M+H]^+^
25	Quercetin hexoside isomer 1	Flavonoid	C21H20O11	464.095	15.30	[M+H]^+^
26	Quercetin hexoside isomer 2	Flavonoid	C21H20O11	464.0955	16.16	[M+H]^+^
27	Quercetin O-pentoside	Flavonoid	C21H20O10	434.0845	18.51	[M+H]^+^
28	Quercetin	Flavonoid	C15H10O7	302.0423	18.51	[M+H]^+^
29	Unknown *m*/*z* 198.1255			198.1255	24.69	[M+H]^+^
30	Phytosphingosine	Sphingolipid	C18H37NO2	317.293	26.45	[M+H]^+^
31	Sphingosine	Sphingolipid	C18H37NO	274.1939	26.77	[M+H]^+^
32	Hydroxy-trimethoxyflavanone	Flavonoid	C17H18O7	328.0958	27.14	[M+H]^+^
33	Methyldioctylamine	Amine	C16H35N	255.2925	28.12	[M+H]^+^

**Table 4:** Antioxidant properties of the tested extracts *.

Extracts	DPPH (mg TE/g)	ABTS (mg TE/g)	CUPRAC (mg TE/g)	FRAP (mg TE/g)	Chelating (mg EDTAE/g)	PBD (mmol TE/g)
Ethyl acetate	13.32 ± 0.56 ^c^	37.61 ± 4.54 ^c^	85.30 ± 2.96 ^c^	36.55 ± 0.18 ^c^	12.09 ± 0.41 ^a^	2.04 ± 0.13 ^c^
Methanol	892.22 ± 11.52 ^a^	916.21 ± 22.84 ^a^	1082.69 ± 75.57 ^a^	615.05 ± 8.64 ^a^	5.99 ± 0.95 ^c^	3.47 ± 0.16 ^a^
Water (infused)	835.79 ± 25.58 ^b^	786.25 ± 15.34 ^b^	688.95 ± 5.88 ^b^	360.35 ± 7.17 ^b^	9.10 ± 0.83 ^b^	2.31 ± 0.01 ^b^

* Values are reported as mean ± SD of three parallel measurements. PBD: phosphomolybdenum; MCA: metal-chelating activity; TE: Trolox equivalent; EDTAE: EDTA equivalent. Different letters indicate significant differences between the tested extracts (*p* < 0.05).

**Table 5:** Enzyme-inhibitory properties of the tested extracts *.

Extracts	AChE (mg GALAE/g)	BChE (mg GALAE/g)	Tyrosinase (mg KAE/g)	Amylase (mmol ACAE/g)	Glucosidase (mmol ACAE/g)
Ethyl acetate	2.17 ± 0.28 ^ab^	3.21 ± 0.22 ^a^	34.40 ± 1.63 ^c^	0.55 ± 0.02 ^b^	na
Methanol	2.54 ± 0.06 ^a^	1.99 ± 0.32 ^c^	70.15 ± 1.00 ^a^	0.39 ± 0.03 ^c^	1.02 ± 0.02 ^b^
Water (infused)	1.97 ± 0.05 ^b^	2.23 ± 0.15 ^b^	52.85 ± 0.54 ^b^	0.62 ± 0.01 ^a^	1.06 ± 0.01 ^a^

* Values are reported as mean ± SD of three parallel measurements. GALAE: galantamine equivalent; KAE: kojic acid equivalent; ACAE: acarbose equivalent; na: not active. Different letters indicate significant differences between the tested extracts (*p* < 0.05).

**Table 6:** Cytotoxic effects of *P. istanbulicum* extracts on cancer and normal cell lines (IC_50_ (µg/mL)).

Cells	HGC-27	MDA-MB-231	HELA	HT-29	DU-145
Ethyl acetate	92.11	56.23	113.80	60.01	93.48
Methanol	62.96	72.48	57.12	65.82	38.09
Water	29.21	51.05	85.31	45.20	33.28

## Data Availability

The raw data supporting the conclusions of this article will be made available by the authors upon request.

## Supplementary Materials

The following supporting information can be downloaded at https://www.mdpi.com/article/10.3390/plants13233421/s1, Table S1: Relevant protein and enzyme target coordinates of the docking box; Table S2: Relevant protein and enzyme result of the docking scores; Table S3: The docking score (kcal/mol) and interacting residues of the enzyme and protein; Table S4: Selected protein–ligand complexes for MM/PBSA binding free energy analysis based on molecular dynamics simulations.
