# Supplementary material for: Using In Vitro and In Silico Analysis to Investigate the Chemical Profile and Biological Properties of Polygonum istanbulicum Extracts
Source: Plants (Basel). 2024 Dec 5;13(23):3421. doi: 10.3390/plants13233421 (PMC11644544; doi:10.3390/plants13233421)
Supplement: Supplementary file 1 [file plants-13-03421-s001.zip › plants-3336301-supplementary.pdf]

LC-MS-qTOF metabolomic analysis

A metabolomic analysis was conducted using an Agilent 1290 Infinity II system paired with an Agilent 6546 LC/MS QTOF instrument (Agilent, USA). Chromatographic separation was carried out on an Agilent InfinityLab Poroshell 120 EC-C18 column (2 x 150 mm, 2.7 µm). The mobile phase consisted of 0.1% formic acid in water (solvent A) and methanol. The gradient profile was as follows: 0–4 minutes at 85% A, 4–7 minutes at 75% A, 7–9 minutes at 68% A, 9–16 minutes at 60% A, 16–22 minutes at 45% A, and 22–28 minutes at 5% A, followed by 2 minutes at 5% A. The flow rate was maintained at 0.5 mL/min with a column temperature of 35 °C, and 1 µL of sample was injected.

Both positive and negative ionization modes were employed. The QTOF parameters included a scan range of 100–1000 m/z, drying gas temperature at 160 °C, sheath gas flow rate at 12.0 L/min, sheath gas temperature at 400 °C, capillary voltage of 5.0 kV, nozzle voltage of 2.0 kV, fragmentor voltage set to 140 V, and collision energies of 10, 20, and 40 eV. For MS/MS analysis, the scan range was 50–800 m/z, with a retention time window of 0.5 minutes, an isolation window of 1.3 amu, and an acquisition rate of 3 spectra per second. Two reference masses (112.9855 m/z and 966.0007 m/z) were used for continuous mass correction.

Data processing, including feature extraction and chromatographic alignment, was performed using Mass Hunter Profinder 10.0 software. The alignment parameters were set to a minimum intensity of 1000 counts and a maximum retention time difference of 0.5 minutes. For feature extraction, the parameters were set to an m/z range of 100–1000, a retention time error tolerance of 0.25 minutes, and a mass error of 2 mDa.

Statistical analysis was conducted using the online platform MetaboAnalyst (https://www.metaboanalyst.ca/). Partial least squares-discriminant analysis (PLS-DA) was applied to differentiate the samples, and variable importance in projection (VIP) plots were generated for both ionization modes.

For the identification of key metabolites, targeted MS/MS was performed, and the results were compared with the Metline Database, an internal library, and relevant literature based on retention times and MS/MS fragmentation patterns.

Table S1. Relevant protein and enzyme target coordinates of the docking box.

Group	Target	PDB ID	Grid size X, Y, Z	X, Y, Z dimensions	Reference
Enzyme	*AChE*	2y2v	22 Å X 30 Å X 40 Å	31.062, 20.311, 11.947	(Yagi et al., 2024}
	BChE	3djy	30 Å X 30 Å X 30 Å	44.794, -19.63, -25.227	(Duran et al., 2024)
	Tyr	5m8o	26 Å X 26 Å X 28 Å	-13.194, 5.341, -26.28	(Yagi et al., 2024
	Amylase	2qv4	28 Å 28 Å X 24 Å	14.188, 48.964, 22.886	(Yagi et al., 2024)
	Glucosidase	3w37	42 Å 52 Å X 54 Å	3.091, −8.008, −4.08	(Duran et al., 2024)
**Cancer-related protein and enzyme**	*TP53*	6mxy	28 Å X 40 Å X 24 Å	-11.077, 23.211, -6.103	(Praveen et al., 2024)
	PTEN	1d5r	88 Å X 54 Å X 124 Å	37.029, 78.446, 31.742	(Mondal et al., 2020)
	EGFR	1m17	64 Å X 34 Å X 28 Å	25.126,1.027, 51.326	Center of inhibitor
	ARO	3eqm	40 Å X 44 Å X 40 Å	85.567, 52.652, 44.357	Center of inhibitor
	Eg5	4a5y	40 Å X 40 Å X 40 Å	13.847, 43.528, 42.113	Center of inhibitor
	PI3K delta	4xe0	40 Å X 40 Å X 40 Å	-5.632, -11.8, 21.568	Center of inhibitor
	E2F1	2aze	40 Å X 40 Å X 40 Å	52.322, 24.479, 36.289	(Yu et al., 2010)
	c-Fos	1fos	62 Å X 76 Å X 64 Å	55.088, -4.748, -8.684	(Kumar et al., 2020)
	IFN-γ	1fg9	70 Å X 60 Å X 78 Å	33.23, 1.815, 6.433	(Yu et al., 2010)
	IL-2	2erj	40 Å X 46 Å X 44 Å	23.659, 54.714, -17.097	(Yu et al., 2010)
	IRS-1	1irs	106 Å X 106 Å X 106 Å	-0.325, -0.542, -0.406	(Yu et al., 2010)
	TGF-β1	5vqp	94 Å X 64 Å X 96 Å	80.405, 44.585, 34.318	(Sun et al., 2023)
	CDK4	2w96	42 Å X 64 Å X 74 Å	5.126, -5.071, 74.401	(Karthick et al., 2024)
	Cyclin D1	2w99	26 Å X 30 Å X 26 Å	15.991, 26.615, 49.003	(Yu et al., 2010)
	CDKN1A	5e0u	58 Å X 48 Å X 64 Å	-4.204, 52.009, -8.555	Center of inhibitor

NF-κB p65	5u4k	30 Å X 22 Å X 24 Å	6.313, 13.606, -7.004	(Yu et al., 2010)
BCL-2	6o0k	28 Å X 24 Å X 54 Å	-13.787, 2.227, -11.244	(Zhang et al., 2024)
BCL-W	2y6w	72 Å X 96 Å X 62 Å	-27.006, 5.973, -5.582	(Taghizadeh et al., 2022)
MCL-1	5fdo	40 Å X 50 Å X 40 Å	9.178, 25.395, -6.703	Center of inhibitor
AKT-1	4gv1	20 Å X 38 Å X 34 Å	-17.903, 0.789, 15.945	(Yua et al., 2022)
BRAF	5vam	40 Å X 40 Å X 40 Å	-24.533, 43.972, 43.972	Center of inhibitor
AR	5t8e	68 Å X 90 Å X 54 Å	20.097, 5.256, 10.86	(Gupta et al., 2024)
TOP2B	3qx3	80 Å X 80 Å X 80 Å	27.436, 96.023, 75.157	(Setiawan et al., 2017)
cIAP1-BIR3	4kmn	50 Å X 50 Å X 50 Å	0.277, 53.967, 13.906	Center of inhibitor
MDM2	4wt2	40 Å X 50 Å X 40 Å	8.697, -28.139, -5.25	Center of inhibitor
TUBA1B	4o2b	36 Å X 24 Å X 54 Å	14.76,47.586,15.722	(Yu et al., 2010)
CDK2	6gue	40 Å X 40 Å X 40 Å	-6.463, -24.101, 25.309	Center of inhibitor
TRAF2	2x7f	40 Å X 40 Å X 40 Å	25.769, -5.064, 58.411	Center of inhibitor
Casp-3	3gjq	20 Å X 20 Å X 20 Å	28.642, 34.169, 12.047	(Aboul-Soud et al., 2022)

Table S2. Relevant protein and enzyme result of the docking scores

Group	Compound	PDB ID	Binding energy	PDB ID	Binding energy	PDB ID	Binding energy	PDB ID	Binding energy	PDB ID	Binding energy
Enzyme	*3,4,5,7-Tetramethoxyflavone*	2qv4	-7.9	3w37	-7.1	2y2v	-9.4	3djy	-8.5	5m8o	-6.5
	Quercetin 4-O-glucoside	2qv4	-8.9	3w37	-8.6	2y2v	-11.0	3djy	-9.1	5m8o	-7.7
	Quercetin 3-O-xyloside	2qv4	-8.6	3w37	-8.2	2y2v	-10.5	3djy	-9.8	5m8o	-7.8
	5-Caffeoylquinic acid	2qv4	-8.2	3w37	-8.3	2y2v	-9.3	3djy	-8.5	5m8o	-7.4
	Myricetin 3-O-rhamnoside	2qv4	-10.2	3w37	-9.4	2y2v	-10.2	3djy	-10.6	5m8o	-8.2
	4-Caffeoylquinic acid	2qv4	-8.2	3w37	-7.9	2y2v	-9.4	3djy	-8.0	5m8o	-7.8
	Quercetin 3-O-rutinoside	2qv4	-9.1	3w37	-9.1	2y2v	-10.0	3djy	-10.6	5m8o	-8.8
	Quercetin 3-O-arabinoside	2qv4	-8.6	3w37	-8.2	2y2v	-9.9	3djy	-9.7	5m8o	-7.8
	Quercetin 3-O-galactoside	2qv4	-8.9	3w37	-7.5	2y2v	-11.0	3djy	-9.9	5m8o	-7.6
	Myricetin 3-O-rutinoside	2qv4	-9.9	3w37	-8.4	2y2v	-10.0	3djy	-10.8	5m8o	-8.5
	Laricitrin	2qv4	-8.9	3w37	-8.2	2y2v	-8.7	3djy	-9.4	5m8o	-7.1
	Myricetin 3-O-glucoside	2qv4	-9.0	3w37	-8.1	2y2v	-9.7	3djy	-10.2	5m8o	-7.7
	Chlorogenic acid	2qv4	-8.2	3w37	-7.9	2y2v	-9.4	3djy	-8.3	5m8o	-7.7
	3,5-Dicaffeoylquinic acid	2qv4	-8.7	3w37	-10.0	2y2v	-10.4	3djy	-10.0	5m8o	-8.3
	Myricetin	2qv4	-9.0	3w37	-8.4	2y2v	-10.2	3djy	-9.4	5m8o	-7.5
	Quercetin 3-O-rhamnoside	2qv4	-9.6	3w37	-8.0	2y2v	-11.2	3djy	-10.3	5m8o	-7.8
	Myricetin 3-beta-O-galactoside	2qv4	-8.3	3w37	-8.0	2y2v	-9.7	3djy	-10.0	5m8o	-7.5
	Quercetin 3-O-glucoside	2qv4	-8.9	3w37	-7.7	2y2v	-9.8	3djy	-10.1	5m8o	-7.6

	Hydroxyluteolin	2qv4	-8.8	3w37	-7.9	2y2v	-10.1	3djy	-8.4	5m8o	-7.2
	Syringetin	2qv4	-8.1	3w37	-7.5	2y2v	-9.0	3djy	-8.7	5m8o	-6.7
	3,4-Dicaffeoylquinic acid	2qv4	-8.8	3w37	-9.4	2y2v	-10.4	3djy	-10.5	5m8o	-6.9
MDA-MB-231	*3,4,5,7-Tetramethoxyflavone*	1m17	-8.1	3eqm	-8.4	4a5y	-8.1	4xe0	-7.6		
	Quercetin 4-O-glucoside	1m17	-9.3	3eqm	-8.9	4a5y	-8.2	4xe0	-9.2		
	Quercetin 3-O-xyloside	1m17	-9.3	3eqm	-9.3	4a5y	-8.9	4xe0	-8.2		
	5-Caffeoylquinic acid	1m17	-8.8	3eqm	-8.1	4a5y	-8.4	4xe0	-8.7		
	Myricetin 3-O-rhamnoside	1m17	-10.0	3eqm	-9.9	4a5y	-9.2	4xe0	-9.8		
	4-Caffeoylquinic acid	1m17	-8.1	3eqm	-8.1	4a5y	-8.6	4xe0	-8.9		
	Quercetin 3-O-rutinoside	1m17	-9.9	3eqm	-10.6	4a5y	-9.2	4xe0	-9.7		
	Quercetin 3-O-arabinoside	1m17	-9.3	3eqm	-9.3	4a5y	-9.0	4xe0	-8.2		
	Quercetin 3-O-galactoside	1m17	-9.1	3eqm	-9.2	4a5y	-8.9	4xe0	-8.1		
	Myricetin 3-O-rutinoside	1m17	-10.2	3eqm	-10.5	4a5y	-9.4	4xe0	-9.5		
	Laricitrin	1m17	-8.7	3eqm	-7.9	4a5y	-8.1	4xe0	-8.0		
	Myricetin 3-O-glucoside	1m17	-9.3	3eqm	-8.9	4a5y	-8.7	4xe0	-8.3		
	Chlorogenic acid	1m17	-7.8	3eqm	-8.3	4a5y	-8.5	4xe0	-8.8		
	3,5-Dicaffeoylquinic acid	1m17	-8.7	3eqm	-9.5	4a5y	-8.5	4xe0	-9.5		
	Myricetin	1m17	-8.9	3eqm	-8.2	4a5y	-8.3	4xe0	-8.0		
	Quercetin 3-O-rhamnoside	1m17	-9.7	3eqm	-9.7	4a5y	-9.2	4xe0	-8.2		
	Myricetin 3-beta-O-galactoside	1m17	-9.2	3eqm	-9.2	4a5y	-8.4	4xe0	-8.6		
	Quercetin 3-O-glucoside	1m17	-9.1	3eqm	-9.3	4a5y	-8.9	4xe0	-8.1		
	Hydroxyluteolin	1m17	-8.5	3eqm	-8.3	4a5y	-8.5	4xe0	-8.0		
	Syringetin	1m17	-8.9	3eqm	-8.2	4a5y	-8.0	4xe0	-8.1		

	3,4-Dicaffeoylquinic acid	1m17	-9.1	3eqm	-9.7	4a5y	-8.6	4xe0	-8.4		
HeLa	*3,4,5,7-Tetramethoxyflavone*	6o0k	-6.9	2y6w	-6.5	5fdo	-8.0	4vg1	-8.1	5vam	-5.8
	Quercetin 4-O-glucoside	6o0k	-7.6	2y6w	-7.3	5fdo	-7.2	4vg1	-8.8	5vam	-6.3
	Quercetin 3-O-xyloside	6o0k	-8.0	2y6w	-6.9	5fdo	-7.4	4vg1	-8.3	5vam	-6.1
	5-Caffeoylquinic acid	6o0k	-7.3	2y6w	-6.5	5fdo	-7.2	4vg1	-7.9	5vam	-6.3
	Myricetin 3-O-rhamnoside	6o0k	-8.7	2y6w	-8.0	5fdo	-7.7	4vg1	-9.8	5vam	-6.7
	4-Caffeoylquinic acid	6o0k	-7.1	2y6w	-6.5	5fdo	-7.1	4vg1	-8.2	5vam	-5.9
	Quercetin 3-O-rutinoside	6o0k	-8.3	2y6w	-7.7	5fdo	-7.9	4vg1	-9.3	5vam	-6.0
	Quercetin 3-O-arabinoside	6o0k	-7.9	2y6w	-6.9	5fdo	-7.5	4vg1	-8.6	5vam	-5.9
	Quercetin 3-O-galactoside	6o0k	-7.3	2y6w	-6.8	5fdo	-7.3	4vg1	-8.1	5vam	-6.2
	Myricetin 3-O-rutinoside	6o0k	-8.0	2y6w	-8.2	5fdo	-7.7	4vg1	-9.0	5vam	-6.2
	Laricitrin	6o0k	-7.1	2y6w	-6.5	5fdo	-7.9	4vg1	-8.6	5vam	-5.7
	Myricetin 3-O-glucoside	6o0k	-7.0	2y6w	-7.0	5fdo	-6.3	4vg1	-8.1	5vam	-6.3
	Chlorogenic acid	6o0k	-7.0	2y6w	-6.9	5fdo	-7.5	4vg1	-8.4	5vam	-5.9
	3,5-Dicaffeoylquinic acid	6o0k	-8.7	2y6w	-7.7	5fdo	-8.2	4vg1	-10.3	5vam	-5.7
	Myricetin	6o0k	-7.0	2y6w	-6.5	5fdo	-7.7	4vg1	-8.3	5vam	-5.7
	Quercetin 3-O-rhamnoside	6o0k	-7.5	2y6w	-7.2	5fdo	-7.6	4vg1	-8.0	5vam	-6.0
	Myricetin 3-beta-O-galactoside	6o0k	-7.1	2y6w	-7.0	5fdo	-6.7	4vg1	-8.3	5vam	-5.9
	Quercetin 3-O-glucoside	6o0k	-7.3	2y6w	-6.9	5fdo	-7.2	4vg1	-8.2	5vam	-6.2
	Hydroxyluteolin	6o0k	-7.4	2y6w	-6.7	5fdo	-7.7	4vg1	-7.8	5vam	-5.9
	Syringetin	6o0k	-7.0	2y6w	-6.4	5fdo	-7.6	4vg1	-8.2	5vam	-5.5
	3,4-Dicaffeoylquinic acid	6o0k	-7.3	2y6w	-7.2	5fdo	-8.0	4vg1	-8.6	5vam	-5.7
HGC-27	*3,4,5,7-Tetramethoxyflavone*	4vg1	-8.1	6gue	-8.4	2x7f	-8.9	6o0k	-6.9	3gjq	-7.1

	Quercetin 4-O-glucoside	4vg1	-8.8	6gue	-9.3	2x7f	-9.4	6o0k	-7.6	3gjq	-7.5
	Quercetin 3-O-xyloside	4vg1	-8.3	6gue	-9.4	2x7f	-8.3	6o0k	-8.0	3gjq	-8.1
	5-Caffeoylquinic acid	4vg1	-7.9	6gue	-8.4	2x7f	-7.8	6o0k	-7.3	3gjq	-6.8
	Myricetin 3-O-rhamnoside	4vg1	-9.8	6gue	-9.3	2x7f	-9.6	6o0k	-8.7	3gjq	-8.1
	4-Caffeoylquinic acid	4vg1	-8.2	6gue	-8.3	2x7f	-8.7	6o0k	-7.1	3gjq	-7.4
	Quercetin 3-O-rutinoside	4vg1	-9.3	6gue	-8.9	2x7f	-10.0	6o0k	-8.3	3gjq	-9.0
	Quercetin 3-O-arabinoside	4vg1	-8.6	6gue	-9.5	2x7f	-8.4	6o0k	-7.9	3gjq	-8.2
	Quercetin 3-O-galactoside	4vg1	-8.1	6gue	-9.2	2x7f	-8.3	6o0k	-7.3	3gjq	-7.6
	Myricetin 3-O-rutinoside	4vg1	-9.0	6gue	-9.0	2x7f	-9.2	6o0k	-8.0	3gjq	-8.4
	Laricitrin	4vg1	-8.6	6gue	-8.6	2x7f	-8.9	6o0k	-7.1	3gjq	-7.1
	Myricetin 3-O-glucoside	4vg1	-8.1	6gue	-9.3	2x7f	-8.4	6o0k	-7.0	3gjq	-7.6
	Chlorogenic acid	4vg1	-8.4	6gue	-8.2	2x7f	-8.9	6o0k	-7.0	3gjq	-7.0
	3,5-Dicaffeoylquinic acid	4vg1	-10.3	6gue	-9.4	2x7f	-9.7	6o0k	-8.7	3gjq	-8.2
	Myricetin	4vg1	-8.3	6gue	-8.9	2x7f	-8.6	6o0k	-7.0	3gjq	-7.1
	Quercetin 3-O-rhamnoside	4vg1	-8.0	6gue	-9.5	2x7f	-8.1	6o0k	-7.5	3gjq	-8.1
	Myricetin 3-beta-O-galactoside	4vg1	-8.3	6gue	-9.2	2x7f	-8.0	6o0k	-7.1	3gjq	-7.7
	Quercetin 3-O-glucoside	4vg1	-8.2	6gue	-9.2	2x7f	-8.3	6o0k	-7.3	3gjq	-7.6
	Hydroxyluteolin	4vg1	-7.8	6gue	-9.1	2x7f	-8.8	6o0k	-7.4	3gjq	-6.9
	Syringetin	4vg1	-8.2	6gue	-8.6	2x7f	-8.9	6o0k	-7.0	3gjq	-6.8
	3,4-Dicaffeoylquinic acid	4vg1	-8.6	6gue	-8.4	2x7f	-7.5	6o0k	-7.3	3gjq	-7.5
	3,4,5,7-Tetramethoxyflavone	1fos	-5.5	2aze	-5.4	1fg9	-6.7	2erj	-7.3	1irs	-6.3
HT-29	*Quercetin 4-O-glucoside*	1fos	-5.8	2aze	-6.1	1fg9	-7.3	2erj	-8.4	1irs	-7.5
	Quercetin 3-O-xyloside	1fos	-6.1	2aze	-5.7	1fg9	-7.2	2erj	-8.3	1irs	-7.5

5-Caffeoylquinic acid	1fos	-5.1	2aze	-5.9	1fg9	-7.1	2erj	-7.4	1irs	-6.1
Myricetin 3-O-rhamnoside	1fos	-6.2	2aze	-6.3	1fg9	-7.8	2erj	-9.2	1irs	-7.3
4-Caffeoylquinic acid	1fos	-5.5	2aze	-6.0	1fg9	-6.6	2erj	-7.3	1irs	-7.0
Quercetin 3-O-rutinoside	1fos	-6.2	2aze	-6.1	1fg9	-7.6	2erj	-8.3	1irs	-8.0
Quercetin 3-O-arabinoside	1fos	-6.1	2aze	-5.8	1fg9	-7.3	2erj	-8.1	1irs	-7.6
Quercetin 3-O-galactoside	1fos	-5.9	2aze	-5.9	1fg9	-6.9	2erj	-7.7	1irs	-7.5
Myricetin 3-O-rutinoside	1fos	-6.3	2aze	-6.1	1fg9	-7.3	2erj	-8.9	1irs	-7.2
Laricitrin	1fos	-5.4	2aze	-5.4	1fg9	-6.6	2erj	-7.7	1irs	-6.2
Myricetin 3-O-glucoside	1fos	-5.3	2aze	-5.4	1fg9	-6.8	2erj	-7.9	1irs	-7.1
Chlorogenic acid	1fos	-5.2	2aze	-5.9	1fg9	-6.8	2erj	-7.1	1irs	-6.1
3,5-Dicaffeoylquinic acid	1fos	-6.0	2aze	-6.7	1fg9	-8.0	2erj	-8.4	1irs	-6.8
Myricetin	1fos	-5.4	2aze	-5.4	1fg9	-7.7	2erj	-7.6	1irs	-6.1
Quercetin 3-O-rhamnoside	1fos	-6.1	2aze	-5.9	1fg9	-7.2	2erj	-8.0	1irs	-7.4
Myricetin 3-beta-O-galactoside	1fos	-5.4	2aze	-5.6	1fg9	-7.5	2erj	-8.2	1irs	-6.7
Quercetin 3-O-glucoside	1fos	-5.8	2aze	-5.9	1fg9	-6.9	2erj	-7.5	1irs	-7.4
Hydroxyluteolin	1fos	-5.1	2aze	-5.8	1fg9	-7.3	2erj	-7.5	1irs	-6.3
Syringetin	1fos	-5.1	2aze	-5.3	1fg9	-6.7	2erj	-7.4	1irs	-5.9
3,4-Dicaffeoylquinic acid	1fos	-5.4	2aze	-5.9	1fg9	-7.1	2erj	-8.3	1irs	-7.4
3,4,5,7-Tetramethoxyflavone	5vqp	-6.5	2w96	-8.9	2w99	-6.4	5e0u	-6.8	6mxy	-7.0
Quercetin 4-O-glucoside	5vqp	-7.2	2w96	-9.4	2w99	-7.8	5e0u	-7.6	6mxy	-7.5
Quercetin 3-O-xyloside	5vqp	-7.0	2w96	-9.9	2w99	-7.6	5e0u	-7.4	6mxy	-7.0
5-Caffeoylquinic acid	5vqp	-6.4	2w96	-8.6	2w99	-6.7	5e0u	-6.7	6mxy	-6.7
Myricetin 3-O-rhamnoside	5vqp	-7.9	2w96	-9.8	2w99	-8.1	5e0u	-7.9	6mxy	-7.5

4-Caffeoylquinic acid	5vqp	-6.4	2w96	-8.0	2w99	-6.8	5e0u	-6.8	6mxy	-6.7
Quercetin 3-O-rutinoside	5vqp	-7.4	2w96	-9.4	2w99	-8.2	5e0u	-7.8	6mxy	-7.8
Quercetin 3-O-arabinoside	5vqp	-7.2	2w96	-10.1	2w99	-7.7	5e0u	-7.3	6mxy	-7.2
Quercetin 3-O-galactoside	5vqp	-7.2	2w96	-9.4	2w99	-7.4	5e0u	-7.2	6mxy	-6.5
Myricetin 3-O-rutinoside	5vqp	-7.5	2w96	-9.6	2w99	-8.4	5e0u	-7.8	6mxy	-7.6
Laricitrin	5vqp	-6.5	2w96	-9.1	2w99	-7.0	5e0u	-6.9	6mxy	-6.3
Myricetin 3-O-glucoside	5vqp	-7.0	2w96	-9.4	2w99	-7.4	5e0u	-7.7	6mxy	-6.7
Chlorogenic acid	5vqp	-6.6	2w96	-8.4	2w99	-7.0	5e0u	-6.7	6mxy	-7.2
3,5-Dicaffeoylquinic acid	5vqp	-7.7	2w96	-9.0	2w99	-7.3	5e0u	-7.7	6mxy	-8.3
Myricetin	5vqp	-6.6	2w96	-9.1	2w99	-6.9	5e0u	-6.9	6mxy	-6.4
Quercetin 3-O-rhamnoside	5vqp	-7.3	2w96	-9.7	2w99	-7.7	5e0u	-7.6	6mxy	-6.8
Myricetin 3-beta-O-galactoside	5vqp	-7.3	2w96	-9.0	2w99	-7.5	5e0u	-7.6	6mxy	-6.7
Quercetin 3-O-glucoside	5vqp	-7.1	2w96	-9.3	2w99	-7.3	5e0u	-7.2	6mxy	-6.5
Hydroxyluteolin	5vqp	-6.8	2w96	-8.4	2w99	-6.8	5e0u	-7.1	6mxy	-6.6
Syringetin	5vqp	-6.4	2w96	-9.1	2w99	-6.9	5e0u	-7.0	6mxy	-6.5
3,4-Dicaffeoylquinic acid	5vqp	-6.6	2w96	-8.6	2w99	-7.3	5e0u	-7.3	6mxy	-7.1
3,4,5,7-Tetramethoxyflavone	5u4k	-4.1								
Quercetin 4-O-glucoside	5u4k	-5.1								
Quercetin 3-O-xyloside	5u4k	-4.8								
5-Caffeoylquinic acid	5u4k	-4.8								
Myricetin 3-O-rhamnoside	5u4k	-5.4								
4-Caffeoylquinic acid	5u4k	-4.5								
Quercetin 3-O-rutinoside	5u4k	-5.2								

	Quercetin 3-O-arabinoside	5u4k	-4.7				
	Quercetin 3-O-galactoside	5u4k	-4.5				
	Myricetin 3-O-rutinoside	5u4k	-5.2				
	Laricitrin	5u4k	-4.3				
	Myricetin 3-O-glucoside	5u4k	-4.7				
	Chlorogenic acid	5u4k	-5.0				
	3,5-Dicaffeoylquinic acid	5u4k	-4.9				
	Myricetin	5u4k	-4.5				
	Quercetin 3-O-rhamnoside	5u4k	-4.8				
	Myricetin 3-beta-O-galactoside	5u4k	-5.0				
	Quercetin 3-O-glucoside	5u4k	-4.5				
	Hydroxyluteolin	5u4k	-4.4				
	Syringetin	5u4k	-4.2				
	3,4-Dicaffeoylquinic acid	5u4k	-4.4				
DU-145	*3,4,5,7-Tetramethoxyflavone*	5t8e	-7.5	3qx3	-7.9	1d5r	-8.0
	Quercetin 4-O-glucoside	5t8e	-8.1	3qx3	-9.5	1d5r	-8.7
	Quercetin 3-O-xyloside	5t8e	-8.1	3qx3	-8.4	1d5r	-7.8
	5-Caffeoylquinic acid	5t8e	-7.1	3qx3	-8.6	1d5r	-7.3
	Myricetin 3-O-rhamnoside	5t8e	-8.2	3qx3	-9.0	1d5r	-8.6
	4-Caffeoylquinic acid	5t8e	-7.7	3qx3	-7.8	1d5r	-7.7
	Quercetin 3-O-rutinoside	5t8e	-7.8	3qx3	-9.7	1d5r	-9.0
	Quercetin 3-O-arabinoside	5t8e	-7.8	3qx3	-8.6	1d5r	-8.0
	Quercetin 3-O-galactoside	5t8e	-7.6	3qx3	-8.7	1d5r	-7.7

Myricetin 3-O-rutinoside	5t8e	-8.0	3qx3	-9.6	1d5r	-8.9
Laricitrin	5t8e	-7.4	3qx3	-8.1	1d5r	-7.7
Myricetin 3-O-glucoside	5t8e	-7.5	3qx3	-8.8	1d5r	-7.4
Chlorogenic acid	5t8e	-7.2	3qx3	-8.9	1d5r	-7.6
3,5-Dicaffeoylquinic acid	5t8e	-8.6	3qx3	-8.6	1d5r	-8.8
Myricetin	5t8e	-7.3	3qx3	-8.4	1d5r	-7.6
Quercetin 3-O-rhamnoside	5t8e	-8.0	3qx3	-8.8	1d5r	-7.9
Myricetin 3-beta-O-galactoside	5t8e	-8.2	3qx3	-8.8	1d5r	-7.5
Quercetin 3-O-glucoside	5t8e	-7.6	3qx3	-8.7	1d5r	-7.7
Hydroxyluteolin	5t8e	-7.7	3qx3	-8.8	1d5r	-7.8
Syringetin	5t8e	-7.4	3qx3	-7.8	1d5r	-7.5
3,4-Dicaffeoylquinic acid	5t8e	-8.2	3qx3	-9.2	1d5r	-7.8

Table S3. The docking score (kcal/mol) and interacting residues of the enzyme and protein

Compound	Target	PDB ID	Binding energy	RMSD	Interaction		Binding site
					Type	Number	
Quercetin 3-O-rhamnoside	*CDK2*	6gue	-9.5	1.09	H-bond	10	Tyr A:15,Tyr A:15,Lys A:33,Leu A:83,Asp A:86,Asp A:86,Asp A:86,Asp A:145,Asp A:145,Asp A:145
Quercetin 3-O-arabinoside	*CDK2*	6gue	-9.5	0.90	H-bond	10	Ile A:10,Glu A:12,Glu A:12,Tyr A:15,Lys A:33,Leu A:83,Asp A:86,Asp A:86,Lys A:129,Asp A:145
Quercetin 3-O-xyloside	*CDK2*	6gue	-9.4	0.75	H-bond	12	Glu A:12,Glu A:12,Tyr A:15,Lys A:33,Leu A:83,Asp A:86,Asp A:86,Lys A:129,Gln A:131,Asp A:145,Asp A:145
3,5-Dicaffeoylquinic acid	*CDK2*	6gue	-9.4	1.06	H-bond	10	Glu A:12, Tyr A:15,Lys A:33,Leu A:83,Leu A,83,Leu A:83, Leu A:83,Asp A:86,Gln A:131
Quercetin 4-O-glucoside	*CDK2*	6gue	-9.3	0.73	H-bond	3	Tyr A:15,Lys A:33,Asp A:145
Myricetin 3-O-rhamnoside	*CDK2*	6gue	-9.3	0.95	H-bond	10	Ile A:10,Glu A:12,Tyr A:15,Leu A:83,Leu A:83,Asp A:86,Asp A:86,Lys A:129,Asn A:132,Asp A:145
Myricetin 3-O-glucoside	*CDK2*	6gue	-9.3	0.79	H-bond	10	Glu A:12,Tyr A:15,Leu A:83,His A:84,Asp A:86,Asp A:86,Asp A:86,Lys A:129,Gln A:131,Asp A:145
Quercetin 3-O-glucoside	*CDK2*	6gue	-9.2	0.69	H-bond	11	Tyr A:15,Glu A:81,Leu A:83,Leu A:83,Asp A:86,Asp A:86,Asp A:86,Lys A:89,Gln A:131,Asp A:145
Quercetin 3-O-galactoside	*CDK2*	6gue	-9.2	0.67	H-bond	8	Tyr A:15,Leu A:83,Asp A:86,Asp A:86,Asp A:86,Lys A:89,Asp A:145,Asp A:145
Myricetin 3-beta-O-galactoside	*CDK2*	6gue	-9.2	0.71	H-bond	7	Ile A:10,Leu A:83,Asp A:86,Asp A:86,Asp A:86,Lys A:89,Asp A:145
Hydroxyluteolin	*CDK2*	6gue	-9.1	0.79	H-bond	5	Lsy A:33,Leu A:83,Asp A:86,Asp A:86, Lys A:89
Myricetin 3-O-rutinoside	*CDK2*	6gue	-9.0	0.45	H-bond	14	Lys A:9,Glu A:12,Tyr A:15,Lys A:33,His A:84,Asp A:86,Asp A:86,Asp A:86,Lys A:129,Asp A:145,Asp A:145,Asp A:145,Asp A:145
Myricetin 3-O-rhamnoside	*PI3K delta*	4xe0	-9.8	0.22	H-bond	11	Arg A:246,Gln A:610,Leu A:612,Leu A:613,Lys A:642,Leu A:735,Asp A:736,Asp A:736,Gln A:792,Cys A:815,Cys A:815
Quercetin 3-O-rutinoside	*PI3K delta*	4xe0	-9.7	0.21	H-bond	9	Asp A:606,Phe A:609,Gln A:610,Leu A:612,Leu A:613,His A:650,Asp A:736,Gln A:792,Lys A:802
Myricetin 3-O-rutinoside	*PI3K delta*	4xe0	-9.5	0.72	H-bond	22	Arg A:246,Arg A:246,Glu A:248,Gln A:260,Ser A:264,Ser A:264,Asp A:736,Ser A:738,Gln A:795,Lys A:802,Arg A:809,Arg A:809,Thr A:811,Arg A:902,Arg A:902,Gln A:906,Gln A:906,,Lys A:802,Arg A:809,,Arg A:809
3,5-Dicaffeoylquinic acid	*PI3K delta*	4xe0	-9.5	2.79	H-bond	13	Phe A:609,Phe A:609,Leu A:613,His A:650,Leu A:735,Asp A:736,Gln A:792,Gln A:795,Lys A:802,Arg A:809,Met A:810,Gly A:814,Cys A:815
Quercetin 4-O-glucoside	*PI3K delta*	4xe0	-9.2	1.09	H-bond	9	Gln A:260,Phe A:609,Gln A:610,His A:650,Asp A:736,Thr A:739,Leu A:791,Gln A:792,Gly A:814
3,5-Dicaffeoylquinic acid	*AKT*	4vg1	-10.3	0.39	H-bond	9	Gly A:157,Phe A:161,Gly A:162,Ala A:230,Gly A:294,,Lys A:276,,Lys A:179

Myricetin 3-O-rhamnoside	*AKT*	4vg1	-9.8	0.22	H-bond	6	Gly A:157,Lys A:158,Lys A:179,Ala A:230,Ala A:230,Asp A:292
Quercetin 3-O-rutinoside	*AKT*	4vg1	-9.3	0.92	H-bond	10	Lys A:158,Lys A:158,Lys A:179,Glu A:191,His A:194,Glu A:234,Lys A:276,Asn A:279,,Lys A:276
Myricetin 3-O-rutinoside	*AKT*	4vg1	-9.0	3.86	H-bond	9	Phe A:161,Lys A:163,Thr A:291,Asp A:292,Asp A:292,Gly A:294,Leu A:295,,Lys A:276
Myricetin 3-O-rutinoside	*Eg5*	4a5y	-9.4	0.74	H-bond	8	Glu A:118,Arg A:119,Gly A:217,Ala A:218,Arg A:221,Arg A:221,Thr A:222,Ser A:232
Quercetin 3-O-rutinoside	*Eg5*	4a5y	-9.2	7.52	H-bond	11	Thr A:112,Thr A:112,Glu A:116,Arg A:119,Arg A:221,Arg A:221,Ser A:232,,Arg A:221,,Arg A:221
Quercetin 3-O-rhamnoside	*Eg5*	4a5y	-9.2	1.03	H-bond	3	Glu A:116,Glu A:116,Arg A:119
Myricetin 3-O-rhamnoside	*Eg5*	4a5y	-9.2	0.02	H-bond	9	Gly A:117,Glu A:118,Arg A:119,Gly A:134,Glu A:215,,Arg A:119,,Arg A:221
Quercetin 3-O-arabinoside	*Eg5*	4a5y	-9.0	0.48	H-bond	4	Glu A:116,Arg A:119,Arg A:119,Leu A:214
3,5-Dicaffeoylquinic acid	*Glucosidase*	3w37	-10.0	2.10	H-bond	14	Asp A:232,Ala A:234,Tyr A:243,Asp A:469,Asn A:496,Ser A:497,Ser A:497,Ser A:497,Ser A:505,Ser A:505,Arg A:552,Asp A:568,,Lys A:506
Myricetin 3-O-rhamnoside	*Glucosidase*	3w37	-9.4	1.08	H-bond	10	Asn A:417,Lys A:421,Gly A:424,Asp A:440,Asp A:440,Asp A:440,Asp A:443,Asp A:443,,Lys A:421
3,4-Dicaffeoylquinic acid	*Glucosidase*	3w37	-9.4	2.20	H-bond	12	Ala A:234,Asn A:237,Asp A:469,Asn A:496,Asn A:496,Ser A:497,Ser A:497,Ser A:505,Ser A:505,Asp A:568,Asp A:568,His A:626
Quercetin 3-O-rutinoside	*Glucosidase*	3w37	-9.1	0.87	H-bond	10	Ala A:234,Asn A:496,Ser A:497,Ser A:497,Ser A:505,Ser A:505,Lys A:506,Arg A:552,,Lys A:506
Quercetin 3-O-rutinoside	*TOP2A*	3qx3	-9.7	0.71	H-bond	12	Arg A:688,Arg A:689,Ser A:725,Ser A:725,Ile A:731,Ser A:733,Ser A:733,Arg A:743,Arg A:743,His A:775,Glu A:853,Glu A:855
Myricetin 3-O-rutinoside	*TOP2A*	3qx3	-9.6	18.46	H-bond	16	Arg A:688,Arg A:688,Ile A:731,Ser A:733,Ser A:733,Arg A:743,Arg A:743,Lys A:744,His A:774,His A:774,Asp A:847,Asp A:847,Asp A:847,Glu A:853,Glu A:853,Glu A:855
Quercetin 4-O-glucoside	*TOP2A*	3qx3	-9.5	1.04	H-bond	11	Ser A:725,Pro A:732,Lys A:744,His A:775,Glu A:853,Pro A:854,Glu A:855,Trp A:856,Asp A:1020,,Arg A:743
3,4-Dicaffeoylquinic acid	*TOP2A*	3qx3	-9.2	3.00	H-bond	12	Glu A:728,Ser A:733,Ser A:733,Ser A:733,Arg A:743,Arg A:743,Glu A:853,Glu A:853,,Arg A:688,Arg A:688,Arg A:689
Myricetin 3-O-rhamnoside	*TOP2A*	3qx3	-9.0	0.79	H-bond	7	Asn A:790,Asn A:798,Asn A:798,Pro A:802,Thr A:828,Thr A:828,Thr A:980
Quercetin 3-O-rutinoside	*Casp-3*	3gjq	-9.0	1.04	H-bond	12	Thr C:62,Thr C:62,Ser C:63,Ser C:65,Ser D:205,Ser D:205,Ser D:205,Arg D:207,Arg D:207,Ser D:209,Ser D:209,Phe D:250
Quercetin 3-O-rutinoside	*ARO*	3eqm	-10.6	0.88	H-bond	10	Arg A:115,Arg A:115,Trp A:141,Arg A:145,Ala A:306,Thr A:310,Arg A:435,Arg A:435,Ala A:438
Myricetin 3-O-rutinoside	*ARO*	3eqm	-10.5	1.04	H-bond	7	Arg A:115,Arg A:115,Thr A:310,Leu A:372,Pro A:429,,Arg A:115
Myricetin 3-O-rhamnoside	*ARO*	3eqm	-9.9	0.97	H-bond	7	Arg A:115,Ile A:133,Trp A:141,Arg A:145,Thr A:310,Ala A:438,,Arg A:115,Arg A:115,Arg A:145,Arg A:435
Quercetin 3-O-rhamnoside	*ARO*	3eqm	-9.7	0.28	H-bond	9	Arg A:115,Arg A:115,Ile A:132,Trp A:141,Arg A:145,Ala A:306,Thr A:310,Pro A:429,Phe A:430,Arg A:435,Arg A:435,Gly A:436,Ala A:438

3,4-Dicaffeoylquinic acid	*ARO*	3eqm	-9.7	1.05	H-bond	12	Arg A:115,Arg A:115,Ile A:133,Trp A:141,Arg A:145,Ser A:314,Leu A:372,Met A:374,Arg A:435,Arg A:435,,Arg A:115
3,5-Dicaffeoylquinic acid	*ARO*	3eqm	-9.5	1.06	H-bond	11	Arg A:115,Arg A:115,Ile A:133,Trp A:141,Arg A:145,Ser A:314,Leu A:372,Met A:374,Arg A:435,Arg A:435,,Arg A:115
Quercetin 3-O-xyloside	*ARO*	3eqm	-9.3	0.87	H-bond	10	Arg A:115,Trp A:141,Arg A:145,Thr A:310,Ser A:314,Ser A:314,Val A:370,Arg A:435,Gly A:436,Ala A:438
Quercetin 3-O-glucoside	*ARO*	3eqm	-9.3	1.05	H-bond	12	Arg A:115,Arg A:115,Asp A:309,Leu A:372,Met A:374,Pro A:429,Phe A:430,Arg A:435,,Arg A:115
Quercetin 3-O-arabinoside	*ARO*	3eqm	-9.3	0.54	H-bond	10	Arg A:115,Arg A:115,Ile A:132,Trp A:141,Arg A:145,Thr A:310,Pro A:429,Phe A:430,Arg A:435,Arg A:435,Gly A:436,Ala A:438
Quercetin 3-O-galactoside	*ARO*	3eqm	-9.2	1.05	H-bond	13	Arg A:115,Arg A:115,Ile A:132,Trp A:141,Arg A:145,Ala A:306,Thr A:310,Arg A:435,Arg A:435,Ala A:438
Myricetin 3-beta-O-galactoside	*ARO*	3eqm	-9.2	0.40	H-bond	10	Arg A:115,Thr A:310,Thr A:310,Ser A:314,Ser A:314,Phe A:430,Arg A:435
Myricetin 3-O-rutinoside	*BChE*	3djy	-10.8	0.79	H-bond	10	Asp A:70,Trp A:82,Gly A:115,Gly A:116,Gly A:117,Tyr A:128,Ala A:199,Pro A:285,Tyr A:332,His A:438
Quercetin 3-O-rutinoside	*BChE*	3djy	-10.6	0.65	H-bond	9	Asn A:68,Trp A:82,Gly A:115,Gly A:115,Gly A:116,Tyr A:128,Leu A:286,Ser A:287,His A:438
Myricetin 3-O-rhamnoside	*BChE*	3djy	-10.6	1.08	H-bond	6	Asn A:68,Asp A:70,Asp A:70,Trp A:82,Pro A:285,Tyr A:332
3,4-Dicaffeoylquinic acid	*BChE*	3djy	-10.5	0.30	H-bond	13	Asp A:70,Asp A:70,Asp A:70,Asn A:83,Asn A:83,Gly A:115,Gly A:115,Gly A:116,Tyr A:128,Ala A:199,Ser A:224,Tyr A:332,His A:438
Quercetin 3-O-rhamnoside	*BChE*	3djy	-10.3	0.61	H-bond	8	Asp A:70,Trp A:82,Gly A:116,Gly A:117,Tyr A:128,Tyr A:128,Ala A:199,Tyr A:332
Myricetin 3-O-glucoside	*BChE*	3djy	-10.2	0.14	H-bond	10	Asp A:70,Trp A:82,Gly A:115,Gly A:117,Tyr A:128,Glu A:197,Pro A:285,Tyr A:332,Tyr A:332,His A:438
Quercetin 3-O-glucoside	*BChE*	3djy	-10.1	1.00	H-bond	11	Asp A:70,Gly A:116,Gly A:117,Tyr A:128,Tyr A:128,Tyr A:128,Ala A:199,Ala A:199,Tyr A:332,Tyr A:332,Tyr A:332
Myricetin 3-beta-O-galactoside	*BChE*	3djy	-10.0	1.04	H-bond	10	Asp A:70,Trp A:82,Gly A:115,Gly A:115,Tyr A:128,Glu A:197,Ala A:199,Leu A:286,Tyr A:332,His A:438
3,5-Dicaffeoylquinic acid	*BChE*	3djy	-10.0	0.30	H-bond	11	Asp A:70,Asn A:83,Gly A:115,Gly A:115,Gly A:116,Thr A:120,Tyr A:128,Tyr A:128,Ala A:199,Ala A:199,His A:438
Quercetin 3-O-galactoside	*BChE*	3djy	-9.9	0.43	H-bond	10	Trp A:82,Gly A:115,Tyr A:128,Tyr A:128,Glu A:197,Glu A:197,Ala A:199,Ala A:199,Tyr A:332,His A:438
Quercetin 3-O-xyloside	*BChE*	3djy	-9.8	0.68	H-bond	9	Asp A:70,Gly A:115,Gly A:115,Tyr A:128,Glu A:197,Ala A:199,Ala A:199,Tyr A:332,His A:438
Quercetin 3-O-arabinoside	*BChE*	3djy	-9.7	0.53	H-bond	8	Gly A:115,Tyr A:128,Glu A:197,Ala A:199,Leu A:286,Tyr A:332,Tyr A:332,His A:438
Myricetin	*BChE*	3djy	-9.4	0.92	H-bond	7	Asn A:68,Asp A:70,Asp A:70,Asn A:83,Tyr A:332,Tyr A:332,His A:438
Laricitrin	*BChE*	3djy	-9.4	0.65	H-bond	4	Asp A:70,Asp A:70,Tyr A:332,Tyr A:332
Quercetin 4-O-glucoside	*BChE*	3djy	-9.1	0.60	H-bond	8	Asp A:70,Asp A:70,Asp A:70,Gln A:71,Ser A:72,Asn A:83,Tyr A:332,His A:438
Quercetin 3-O-rhamnoside	*AChE*	2y2v	-11.2	1.05	H-bond	11	Gly A:120,Gly A:120,Gly A:121,Tyr A:124,Tyr A:133,Glu A:202,Glu A:202,Ala A:204,Ala A:204,Tyr A:337,His A:447
Quercetin 4-O-glucoside	*AChE*	2y2v	-11.0	0.15	H-bond	8	Trp A:86,Gly A:120,Tyr A:133,Glu A:202,Ile A:294,Arg A:296,Tyr A:341,His A:447

Quercetin 3-O-galactoside	*AChE*	2y2v	-11.0	0.60	H-bond	11	Asp A:74,Gly A:120,Gly A:121,Tyr A:124,Tyr A:133,Tyr A:133,Glu A:202,Glu A:202,Ala A:204,Ser A:229,His A:447
Quercetin 3-O-xyloside	*AChE*	2y2v	-10.5	0.13	H-bond	8	Asp A:74,Thr A:83,Asn A:87,Asn A:87,Ala A:204,Phe A:295,Tyr A:341,His A:447
3,5-Dicaffeoylquinic acid	*AChE*	2y2v	-10.4	0.20	H-bond	11	Asp A:74,Gly A:121,Tyr A:124,Ala A:204,Ala A:204,Ser A:293,Ser A:293,Phe A:295,Arg A:296,Arg A:296,His A:447
3,4-Dicaffeoylquinic acid	*AChE*	2y2v	-10.4	2.40	H-bond	9	Asp A:74,Asn A:87,Gly A:121,Gly A:122,Tyr A:124,Ala A:204,His A:447,,His A:447
Myricetin 3-O-rhamnoside	*AChE*	2y2v	-10.2	1.08	H-bond	4	Trp A:86,Ala A:204,Tyr A:341,His A:447
Myricetin	*AChE*	2y2v	-10.2	0.99	H-bond	8	Gln A:71,Asp A:74,Asn A:87,Gly A:120,Tyr A:124,Tyr A:133,Tyr A:133,His A:447
Hydroxyluteolin	*AChE*	2y2v	-10.1	0.08	H-bond	4	Gly A:121,Gly A:122,Ser A:293,Tyr A:337
Quercetin 3-O-rutinoside	*AChE*	2y2v	-10.0	0.91	H-bond	10	Tyr A:72,Thr A:75,Thr A:75,Leu A:76,Tyr A:124,Ser A:293,Ser A:293,Phe A:295,Arg A:296,Tyr A:341
Myricetin 3-O-rutinoside	*AChE*	2y2v	-10.0	0.80	H-bond	10	Tyr A:72,Asp A:74,Thr A:75,Leu A:76,Tyr A:124,Ser A:293,Phe A:295,Arg A:296,Tyr A:337,Tyr A:341
Quercetin 3-O-arabinoside	*AChE*	2y2v	-9.9	6.69	H-bond	9	Gly A:121,Gly A:122,Ser A:125,Phe A:295,Arg A:296,Arg A:296,Tyr A:341,Tyr A:341,His A:447
Quercetin 3-O-glucoside	*AChE*	2y2v	-9.8	0.00	H-bond	12	Gly A:121,Gly A:122,Tyr A:124,Tyr A:124,Ser A:125,Tyr A:133,Tyr A:133,Phe A:295,Arg A:296,Arg A:296,Tyr A:341,His A:447
Myricetin 3-O-glucoside	*AChE*	2y2v	-9.7	0.41	H-bond	11	Asp A:74,Trp A:86,Trp A:86,Gly A:120,Gly A:121,Gly A:122,Tyr A:124,Tyr A:133,Phe A:295,Tyr A:341,His A:447
Myricetin 3-beta-O-galactoside	*AChE*	2y2v	-9.7	0.70	H-bond	12	Asn A:87,Gly A:121,Gly A:122,Tyr A:124,Ser A:125,Gly A:126,Ala A:204,Phe A:295,Arg A:296,Arg A:296,Tyr A:341,His A:447
Chlorogenic acid	*AChE*	2y2v	-9.4	0.30	H-bond	9	Asp A:74,Gly A:121,Gly A:122,Ala A:204,Ala A:204,Ser A:293,Phe A:295,Arg A:296,His A:447
4-Caffeoylquinic acid	*AChE*	2y2v	-9.4	0.80	H-bond	6	Tyr A:124,Glu A:202,Phe A:295,Tyr A:341,,His A:447
3,4,5,7-Tetramethoxyflavone	*AChE*	2y2v	-9.4	3.00	H-bond	2	Gly A:121,Gly A:122
5-Caffeoylquinic acid	*AChE*	2y2v	-9.3	0.60	H-bond	7	Gly A:120,Phe A:295,Arg A:296,Tyr A:337,Tyr A:341,,His A:447
Syringetin	*AChE*	2y2v	-9.0	1.08	H-bond	3	Tyr A:72,Tyr A:72,Phe A:295
Quercetin 3-O-rutinoside	*Traf2*	2x7f	-10.0	1.03	H-bond	6	Cys A:108,Gly A:111,Glu A:163,Lys A:303,His A:305,Thr A:309
3,5-Dicaffeoylquinic acid	*Traf2*	2x7f	-9.7	0.98	H-bond	11	Lys A:54,Gly A:109,Ser A:112,Asp A:115,Asp A:115,Asp A:115,Lys A:303,His A:305,Thr A:309,,Lys A:41
Myricetin 3-O-rhamnoside	*Traf2*	2x7f	-9.6	0.94	H-bond	7	Tyr A:36,Lys A:54,Lys A:54,Cys A:108,Cys A:108,Asp A:115,Asp A:171
Quercetin 4-O-glucoside	*Traf2*	2x7f	-9.4	0.96	H-bond	5	Cys A:108,Gly A:109,Ala A:110,Thr A:309,Lys A:312
Myricetin 3-O-rutinoside	*Traf2*	2x7f	-9.2	1.06	H-bond	4	Asp A:115,Glu A:163,Lys A:303,Lys A:311
Quercetin 3-O-arabinoside	*CDK4*	2w96	-10.1	1.06	H-bond	12	Ala B:16,Lys B:35,His B:95,Asp B:97,Asp B:99,Asp B:99,Glu B:144,Asp B:158,,Arg B:101

Quercetin 3-O-xyloside	*CDK4*	2w96	-9.9	0.43	H-bond	14	Val B:14,Gly B:15,Ala B:16,Tyr B:17,His B:95,Val B:96,Asp B:97,Asp B:99,Asp B:99,Asp B:99,Arg B:101,,Arg B:101,,Lys B:35
Myricetin 3-O-rhamnoside	*CDK4*	2w96	-9.8	0.02	H-bond	12	Gly B:15,Ala B:16,Lys B:35,His B:95,Asp B:99,Asp B:99,Thr B:102,Glu B:144,Asp B:158,,Arg B:101
Quercetin 3-O-rhamnoside	*CDK4*	2w96	-9.7	0.52	H-bond	9	Ala B:16,Lys B:35,His B:95,Asp B:99,Asp B:99,Asp B:158,,Arg B:101
Myricetin 3-O-rutinoside	*CDK4*	2w96	-9.6	0.48	H-bond	12	Gly B:15,Ala B:16,Tyr B:17,Glu B:94,His B:95,Val B:96,Asp B:99,Arg B:101,Lys B:142,Asp B:158,,Lys B:35
Quercetin 4-O-glucoside	*CDK4*	2w96	-9.4	1.08	H-bond	14	Gly B:15,Ala B:16,Tyr B:17,Lys B:35,Asp B:99,Asp B:99,Arg B:101,Thr B:102,Asp B:158,Asp B:158,,Lys B:35,,Lys B:35
Quercetin 3-O-rutinoside	*CDK4*	2w96	-9.4	0.31	H-bond	15	Ile B:12,Lys B:35,Asp B:99,Asp B:99,Asp B:99,Arg B:101,Lys B:142,Asn B:145,Asp B:158
Quercetin 3-O-galactoside	*CDK4*	2w96	-9.4	0.92	H-bond	8	Ala B:16,Tyr B:17,Lys B:35,Glu B:94,Asp B:97,Asp B:99,Asp B:99,Arg B:101,Lys B:142,Asp B:158,,Lys B:35
Myricetin 3-O-glucoside	*CDK4*	2w96	-9.4	0.84	H-bond	11	Ala B:16,Lys B:35,Val B:96,Asp B:99,Asp B:99,Lys B:142,Asn B:145
Quercetin 3-O-glucoside	*CDK4*	2w96	-9.3	0.35	H-bond	10	Ala B:16,Lys B:35,Asp B:99,Asp B:99,Asp B:99,Lys B:142,Asn B:145,Asp B:158,Asp B:158,Asp B:158
Syringetin	*CDK4*	2w96	-9.1	0.77	H-bond	9	Ala B:16,Lys B:35,Val B:96,Asp B:97,Asp B:99,Asp B:99,Arg B:101,Lys B:142,Glu B:144,Asn B:145,Asn B:145,Thr B:177,,Lys B:142
Myricetin	*CDK4*	2w96	-9.1	0.31	H-bond	10	Gly B:15,Ala B:16,Tyr B:17,Lys B:35,Gln B:98,Arg B:101,Arg B:101,Lys B:142,Asp B:158,Asp B:158,,Arg B:101,,Lys B:35
Laricitrin	*CDK4*	2w96	-9.1	0.34	H-bond	7	Ala B:16,Lys B:35,Asp B:99,Lys B:142
Myricetin 3-beta-O-galactoside	*CDK4*	2w96	-9.0	0.49	H-bond	14	Ala B:16,Lys B:35,Asp B:97,Asp B:99,Asp B:99,Arg B:101,Lys B:142,Glu B:144,Thr B:177,Thr B:177,,Arg B:101
3,4,5,7-Tetramethoxyflavone	*CDK4*	2w96	-9.0	0.05	H-bond	4	Ala B:16,Tyr B:17,Glu B:94,His B:95,Val B:96,Val B:96,Arg B:101,Asn B:145,Asp B:158,Thr B:177,Trp B:179,,Lys B:35,Arg B:101,Lys B:142,Lys B:142
Myricetin 3-O-rhamnoside	*Amylase*	2qv4	-10.2	0.40	H-bond	10	Tyr A:62,Gln A:63,His A:101,Tyr A:151,Arg A:195,Ala A:198,His A:201,Glu A:233,Glu A:233,His A:299
Myricetin 3-O-rutinoside	*Amylase*	2qv4	-9.9	0.85	H-bond	13	Gln A:63,Asp A:197,Ala A:198,Lys A:200,Glu A:233,Ile A:235,His A:299,Asp A:300,Asp A:300,His A:305,,His A:201,His A:305
Quercetin 3-O-rhamnoside	*Amylase*	2qv4	-9.6	0.91	H-bond	8	Arg A:195,Asp A:197,Lys A:200,His A:201,His A:299,Asp A:300,Asp A:300,His A:305
Quercetin 3-O-rutinoside	*Amylase*	2qv4	-9.1	1.13	H-bond	16	Gln A:63,Gln A:63,Arg A:195,Asp A:197,Ala A:198,Ser A:199,Lys A:200,Lys A:200,Glu A:233,Glu A:233,Ile A:235,His A:299,His A:305,,His A:201,His A:305
Myricetin 3-O-glucoside	*Amylase*	2qv4	-9.0	0.81	H-bond	8	Gln A:63,Glu A:233,Glu A:233,His A:299,Asp A:300,His A:305,His A:305
Myricetin	*Amylase*	2qv4	-9.0	0.21	H-bond	6	Tyr A:62,Gln A:63,His A:101,Arg A:195,His A:299,Asp A:300
Myricetin 3-O-rhamnoside	*IL-2*	2erj	-9.2	0.67	H-bond	9	Glu A:106,Thr A:115,Glu A:116,Glu A:116,Arg A:117,Tyr A:119,,Lys A:16,Arg A:117
Myricetin 3-O-rutinoside	*EGFR*	1m17	-10.2	1.09	H-bond	13	Lys A:721,Lys A:721,Glu A:738,Glu A:738,Leu A:764,Thr A:766,Met A:769,Gly A:772,Arg A:817,Asp A:831,Asp A:831,,Lys A:721
Myricetin 3-O-rhamnoside	*EGFR*	1m17	-10.0	0.90	H-bond	9	Ala A:719,Lys A:721,Glu A:738,Thr A:766,Met A:769,Cys A:773,Arg A:817,Asp A:831,Asp A:831

Quercetin 3-O-rutinoside	*EGFR*	1m17	-9.9	0.99	H-bond	9	Lys A:721,Lys A:721,Glu A:738,Leu A:764,Thr A:766,Gly A:772,,Lys A:721,Lys A:721
Quercetin 3-O-rhamnoside	*EGFR*	1m17	-9.7	1.09	H-bond	9	Lys A:721,Lys A:721,Glu A:738,Thr A:766,Met A:769,Gly A:772,Arg A:817,Asn A:818,Thr A:830
Quercetin 4-O-glucoside	*EGFR*	1m17	-9.3	0.99	H-bond	9	Lys A:721,Thr A:766,Thr A:766,Met A:769,Pro A:770,Pro A:770,Gly A:772,Asp A:776,Asp A:831
Quercetin 3-O-xyloside	*EGFR*	1m17	-9.3	1.06	H-bond	9	Lys A:721,Lys A:721,Glu A:738,Glu A:738,Leu A:764,Thr A:766,Gly A:772,Asp A:831,Asp A:831
Quercetin 3-O-arabinoside	*EGFR*	1m17	-9.3	0.77	H-bond	10	Lys A:721,Lys A:721,Glu A:738,Thr A:766,Thr A:766,Gly A:772,Thr A:830,Asp A:831,Asp A:831,Asp A:831
Myricetin 3-O-glucoside	*EGFR*	1m17	-9.3	0.15	H-bond	12	Leu A:694,Lys A:721,Lys A:721,Glu A:738,Glu A:738,Thr A:766,Thr A:766,Met A:769,Gly A:772,Arg A:817,Asp A:831,Asp A:831
Myricetin 3-beta-O-galactoside	*EGFR*	1m17	-9.2	0.58	H-bond	9	Gly A:695,Ala A:719,Lys A:721,Lys A:721,Glu A:738,Thr A:766,Gly A:772,Asp A:831,Asp A:831
Quercetin 3-O-glucoside	*EGFR*	1m17	-9.1	0.34	H-bond	10	Lys A:721,Lys A:721,Glu A:738,Thr A:766,Gly A:772,Thr A:830,Asp A:831,Asp A:831,,Lys A:721
Quercetin 3-O-galactoside	*EGFR*	1m17	-9.1	1.03	H-bond	11	Ala A:719,Lys A:721,Lys A:721,Glu A:738,Thr A:766,Gly A:772,Thr A:830,Asp A:831,Asp A:831,,Lys A:721
3,4-Dicaffeoylquinic acid	*EGFR*	1m17	-9.1	0.87	H-bond	7	Lys A:721,Leu A:764,Met A:769,Met A:769,Arg A:817,,Lys A:721

Table S4. Selected protein-ligand complexes for MM/PBSA binding free energy analysis based on molecular dynamics simulations.

Complex	Frames	VDWAALS	EEL	EGB	ESURF	GGAS	GSOLV	TOTAL
AKT_3-5-Dicaffeoylquinic acid	Average	-41.12	94.96	-68	-6.6	54	-74.89	-21.1
	SD	0.49	3.99	4.21	0.1	4.5	4.14	0.34
	SEM	0.35	2.82	2.98	0.1	3.2	2.93	0.24
ARO_quercetin 3-O-rutinoside	Average	-68.16	-42.87	65	-8.8	-111	56.21	-54.8
	SD	4.56	6.37	4.85	0.4	8.7	4.64	5.19
	SEM	0.45	0.63	0.48	0	0.9	0.46	0.52
ARO_myricetin 3-O-rutinoside	Average	-46.09	79.9	-44	-5.8	34	-49.29	-15.5
	SD	2.58	24.05	23.1	0.3	24	23.14	4.14
	SEM	0.26	2.39	2.3	0	2.4	2.3	0.41
CDK4_quercetin 3-O-arabinoside	Average	-36.13	-60.78	71.1	-6.2	-97	64.9	-32.0
	SD	3.47	10.66	8.24	0.3	10	8.14	3.92
	SEM	0.35	1.06	0.82	0	1	0.81	0.39
EGFR_myricetin 3-O-rhamnoside	Average	-40.76	-56.25	60.7	-6.8	-97	53.94	-43.1
	SD	3.76	7.52	4.04	0.2	6.6	3.95	4.7
	SEM	0.37	0.75	0.4	0	0.7	0.39	0.47
EGFR_myricetin 3-O-rutinoside	Average	-39.38	-52.97	67.3	-6.3	-92	60.99	-31.4
	SD	3.27	9.53	5.56	0.1	6.3	5.68	0.58
	SEM	2.32	6.74	3.93	0.1	4.4	4.02	0.41

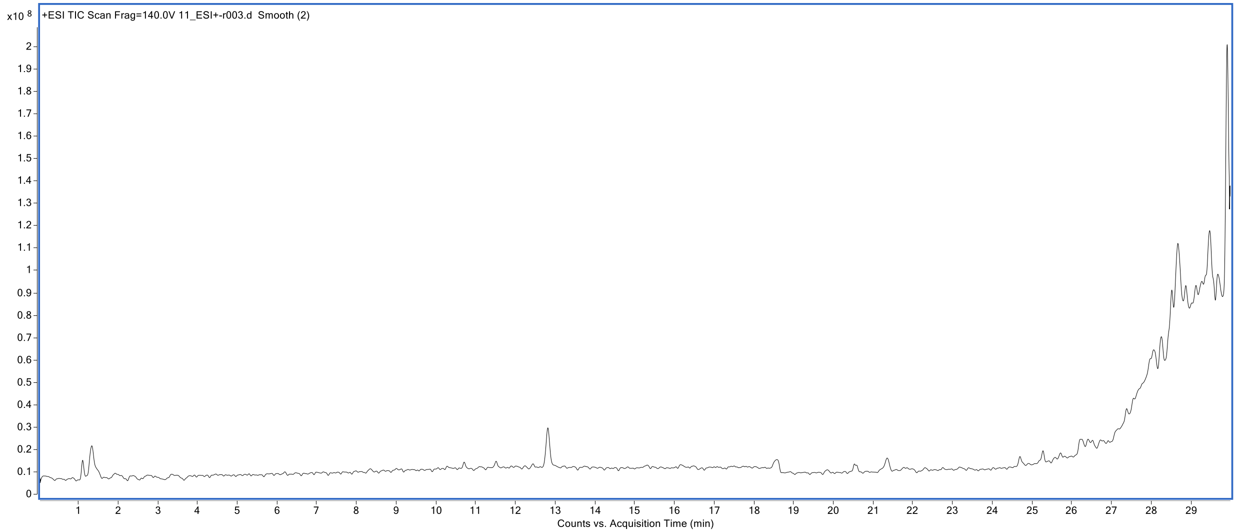

TIC of *Polygonum istanbulicum* of ethyl acetate extract in positive ionization mode

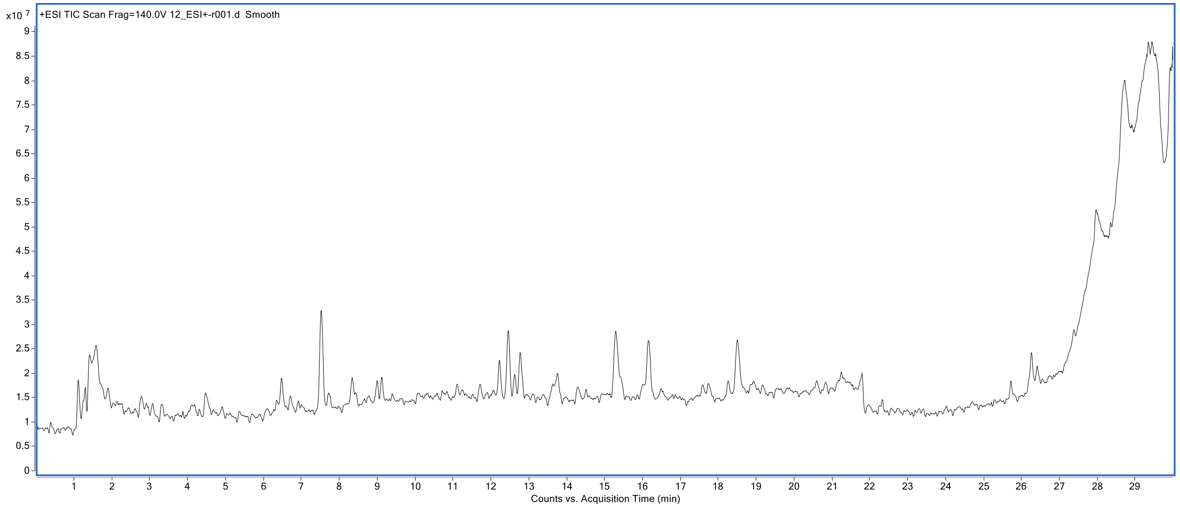

TIC of *Polygonum istanbulicum* of methanol extract in positive ionization mode

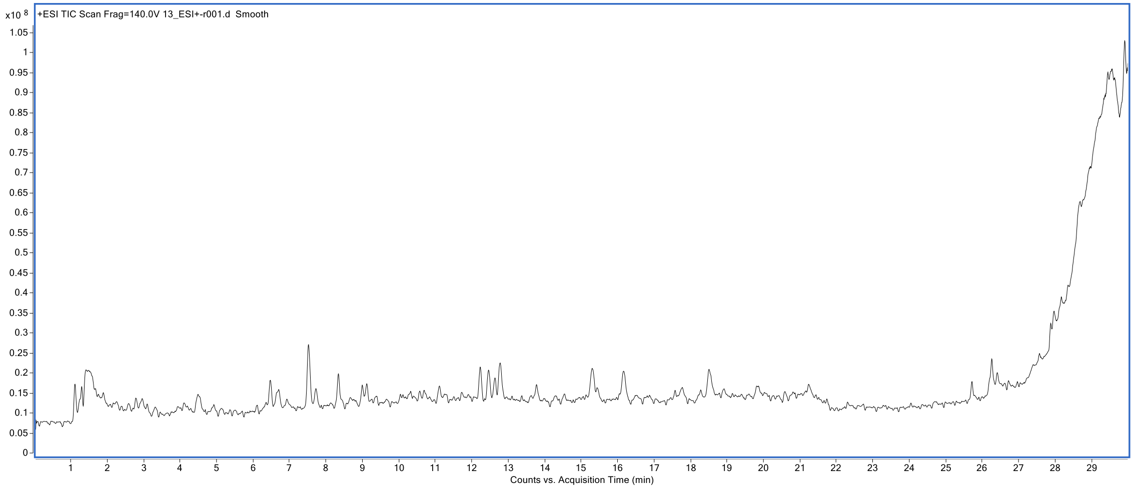

TIC of *Polygonum istanbulicum* of water extract in positive ionization mode

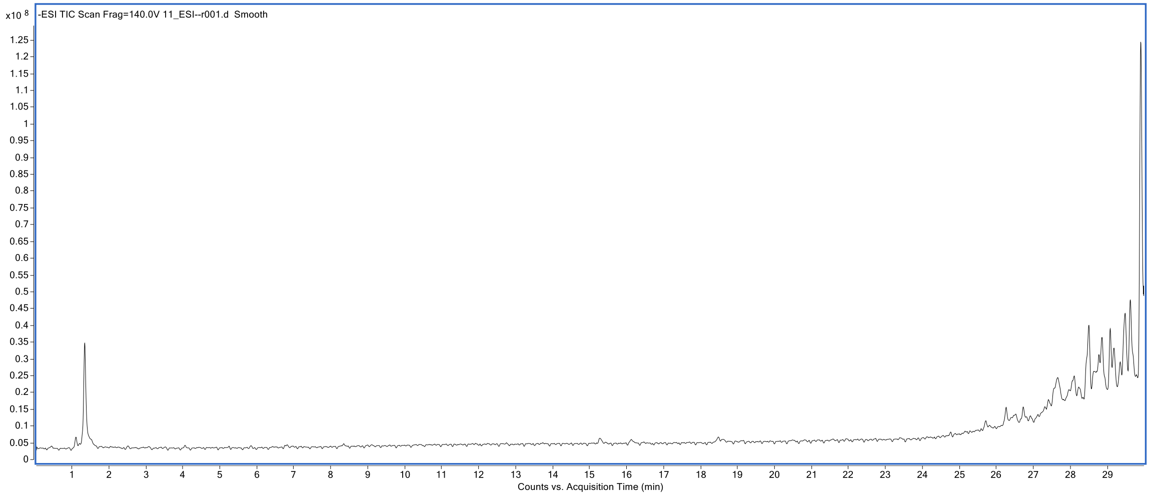

TIC of *Polygonum istanbulicum* of ethyl acetate extract in negative ionization mode

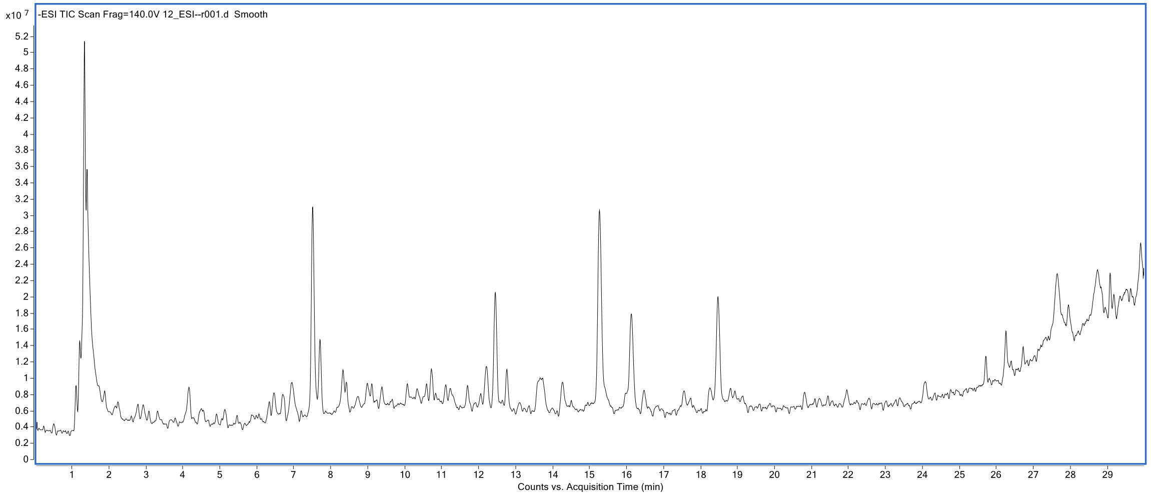

TIC of *Polygonum istanbulicum* of methanol extract in negative ionization mode

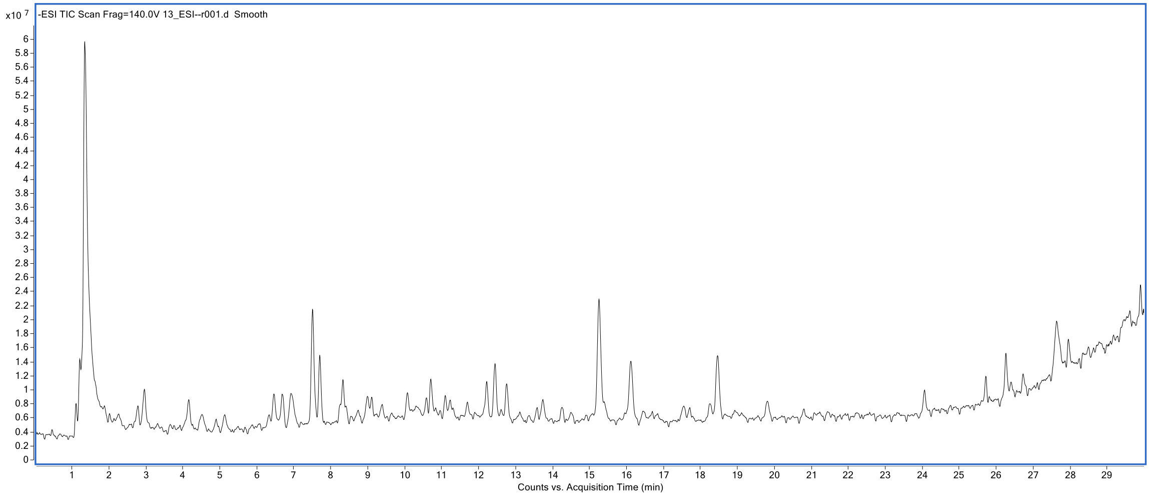

TIC of *Polygonum istanbulicum* of water extract in negative ionization mode
